# Total Synthesis of Mycalisine B

**DOI:** 10.3390/md17040226

**Published:** 2019-04-14

**Authors:** Haixin Ding, Zhizhong Ruan, Peihao Kou, Xiangyou Dong, Jiang Bai, Qiang Xiao

**Affiliations:** Jiangxi Key Laboratory of Organic Chemistry, Institute of Organic Chemistry, Jiangxi Science & Technology Normal University, Nanchang 330013, China; dinghaixin_2010@163.com (H.D.); ruanzhizhong0404@yeah.net (Z.R.); koupeihao92@yeah.net (P.K.); dongxiangyou93@yeah.net (X.D.); mtbaijiang@yeah.net (J.B.)

**Keywords:** marine nucleoside, total synthesis, vorbrüggen glycosylation, mycalisine B

## Abstract

The first total synthesis of the marine nucleoside Mycalisine B—a naturally occurring and structurally distinct 4,5-unsaturated 7-deazapurine nucleoside—has been accomplished in 10 linear steps with 27.5% overall yield from commercially available 1,2,3,5-tetra-*O*-acetyl-ribose and tetracyanoethylene. Key steps of the approach include: (1) I_2_ catalyzed acetonide formation from 1,2,3,5-tetra-*O*-acetylribose and acetone at large scale; (2) Vorbrüggen glycosylation using *N*^4^-benzoyl-5-cyano-6-bromo-7*H*-pyrrolo[2,3–*d*]pyrimidine as a nucleobase to avoid formation of *N*-3 isomer; (3) mild and scalable reaction conditions.

## 1. Introduction

**Figure 1:**
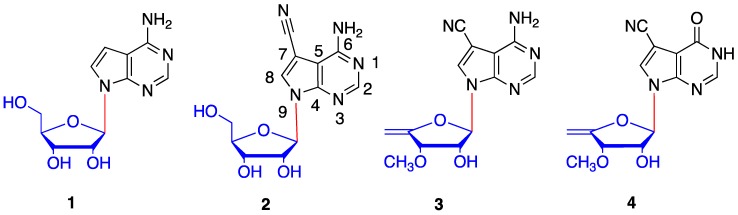
Structures of tubercidin (**1**), toyocamycin (**2**), mycalisine A (**3**) and mycalisine B (**4**).

Naturally occurring compounds, isolated from marine invertebrates, are a valuable and promising resource for the identification of novel drug leads with unprecedented and novel mechanisms of action [1,2,3]. Until now, about 15,000 species of marine sponges—which are the most primitive and simplest multicellular animal—exist worldwide. These marine sponges have produced a wide range of secondary metabolites with diverse structural features and distinct biological activities [4,5,6]. Among these, mycalisines A and B (Figure 1) were isolated from a Japanese sponge *Mycale sp.* in 1985. Biological activities evaluation showed that mycalisines A and B possess cell division inhibition of fertilized starfish eggs at IC_50_ 0.5 and 200 µg/mL, respectively [7]. Mycalisines A and B represent structurally distinct 4′,5′-unsaturated 7-deazapurine nucleosides possessing pyrrolo[2,3-*d*]pyrimidine.

It is noteworthy that a series of naturally occurring 7-deazapurine nucleosides—such as tubercidin **1** [8] and toyocamycin **2** [9] (Figure 1) from *Streptomyces sp*—have been found to display interesting biological activities [10,11,12]. In the structure of 7-deazapurine (pyrrolo[2,3-*d*]pyrimidine) nucleosides, *N*-7 of the purine base is replaced by a carbon atom. Thus, the resulting pyrrole moiety is more electron-rich than the imidazole moiety of the corresponding purine and is thus likely to be more prone to cation–π or π–π interactions with DNA/RNA or proteins [13,14,15]. In the past decade, a number of 7-deazapurine nucleosides have been synthesized and proved to be a privileged scaffold in the design of antitumor and antiviral nucleosides [16]. Moreover, 4’,5’-unsaturated nucleosides possess an exocyclic double bond next to the ring oxygen of ribose. These nucleosides display powerful biopotency and could interfere with some specific metabolic pathways [17,18]. Because of the rich reactivity of the enol ether functional group, they offered many possibilities for further chemical transformations [19].

**Figure 2:**
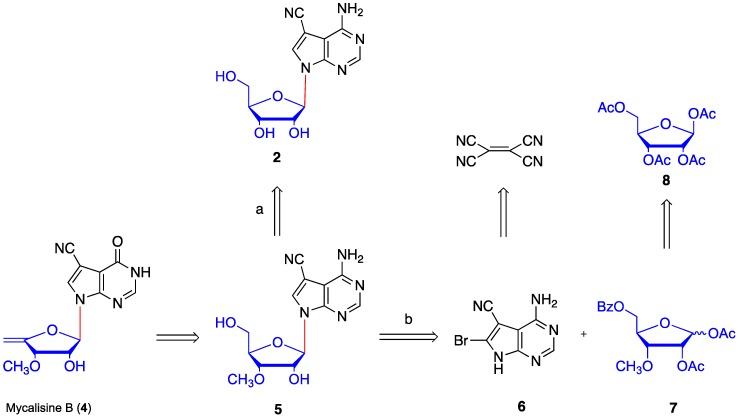
Retrosynthetic analysis of Mycalisine B (**a**) using toyocamycin as the starting material; (**b**) using Vorbrüggen glycosylation as the key synthetic step.

Due to the above-mentioned reasons and our continuing effort to synthesize bioactive marine nucleosides [20,21,22,23,24,25,26,27], we are interested in the total synthesis and structure-activity relationship studies of 4′,5′-unsaturated 7-deazapurine nucleosides as antibiotics. A literature search revealed that the first total synthesis of mycalisine A was accomplished using toyocamycin as a starting material shortly after it was reported [28]. During this synthesis, methylation of toyocamycin with diazomethane was first conducted using SnCl_2_ as a catalyst (Figure 2a) [29]. It afforded a mixture of 2′-*O*-methyl and 3′-*O*-methyl isomers of toyocamycin with the ratio of 3:2 (yield not given). Separation of the obtained regiomers was not achieved even though various purification methods, including silica chromatography, reversed phase HPLC, and recrystallization, were tried. Therefore, the mixture was directly acetylated with acetic anhydride. The acetate of 3′-*O*-methyl isomers can be enriched to give 90% purity by recrystallization. After removing the acetate group with methanolic ammonia, 3′-*O*-methyl toyocamycin was obtained by recrystallization. Subsequent unsaturation of 5′-hydroxyl afforded mycalisine A. However, this approach is not only impractical, but the starting material toyocamycin is also costly.

Afterwards, our group reported an improved total synthesis of mycalisine A using a commercially available d-xylose as a starting material in eleven linear steps with 15% overall yield [27]. Our strategy used the late-stage Vorbrüggen glycosylation as the key step to synthesize the nucleoside. The corresponding ribose glycosylation donor **7** was obtained from 5-*O*-benzoyl-1,2-*O*-isopropylidene-α-d-xylofuranose by Dess-Martin oxidation, stereoselective NaBH_4_ reduction, and methylation. In the following scale-up of the total synthesis, some obstacles arose. First, the synthesis of 1,2-*O*-isopropylidene-α-d-xylofuranose from d-xylose needs two steps and involved a tedious silica chromatography purification to provide crystalline substance. Second, Dess-Martin oxidation was troublesome at a large scale. Third, the yield of the Vorbrüggen glycosylation was moderate and the *N*-3 isomer was formed as a by-product.

**Scheme 1:**
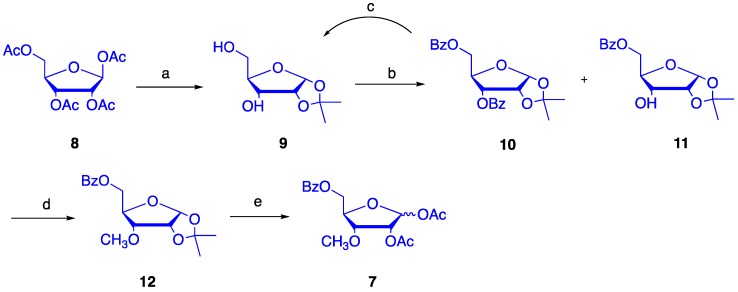
Synthesis of ribose glycosylation donor **7.** Reagents and conditions**:** (**a**) i. I_2_, acetone; ii. K_2_CO_3_, MeOH, r.t., 87%; (**b**) BzCl, E_3_N, CH_2_Cl_2_, **10** 35%, **11** 62%;(**c**) NaOMe, MeOH, r.t. 99%; (**d**) CH_3_I, Ag_2_O, DMF, r.t., 95%; (**e**) Ac_2_O, H_2_SO_4_, HOAc, r.t., 91%.

Therefore, it is necessary to further develop an improved approach for the total synthesis of mycalisines. In the present paper, an expeditious and first total synthesis of mycalisine B was accomplished (Scheme 1) which addressed the corresponding obstacles we have encountered in the synthesis mycalisine A.

## 2. Results and Discussion

According to our retrosynthetic analysis of mycalisine B (Figure 2b), the ribose glycosylation donor **7** can be obtained from 1,2,3,5-tetra-*O*-acetylribose (**8**), which will obviate the oxidation and reduction reactions for using d-xylose as a starting material. 1,2-*O*-acetonide ribose (**9**) was initially synthesized from 1,2,3,5-tetra-*O*-acetylribose (**8**) utilizing AlMe_3_ as catalysis in acetone [30]. The disadvantage of this method is that AlMe_3_ is explosive and difficult to handle. Later, a much-improved protocol was reported which used I_2_ as a catalysis and acetone as solvent to facilitate acetonide-formation [31]. This protocol is exceptionally mild and can be scaled-up smoothly. During our synthesis, 1,2-*O*-acetonide ribose (**9**) was obtained at 100 g scale in two steps and 87% overall yield without purification by a column chromatography (Scheme 1). We also found that the commercially available acetone without drying is qualified for the reaction, which further simplified this protocol.

**Figure 3:**
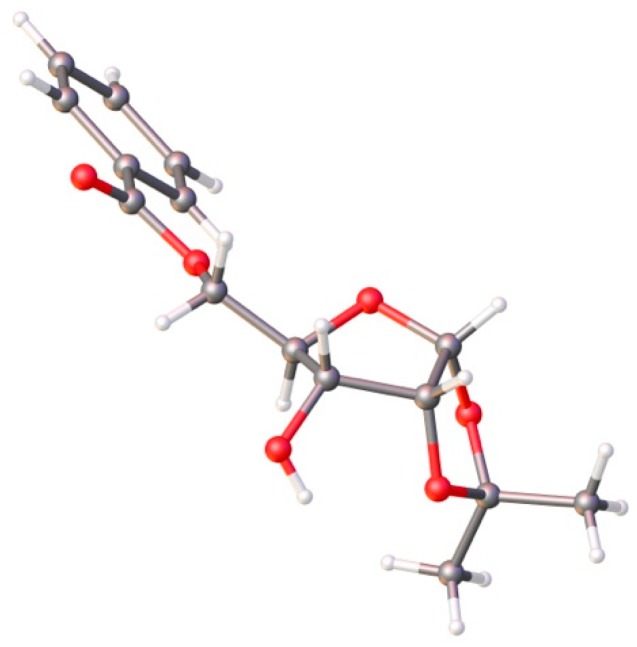
X-ray crystal structure of **11**.

After careful benzoylation at −10 °C, the 5-*O*-benzoyl ribose (**11**) was prepared in 62% yield. Moreover, a single crystal of (**11**), suitable for X-ray crystallography, was obtained and its structure is shown in Figure 3 [32]. Meanwhile, 3,5-*O*-dibenzoyl ribose (**10**) was obtained in 35% yield, which can be deprotected with Zemplén transesterification (catalytic sodium methoxide in methanol) [33] to give 1,2-*O*-acetonide ribose (**9**) in almost quantitative yield. Therefore, the yield increased to 84% following two rounds of this procedure. Subsequently, methylation of 3-OH to synthesize 3-*O*-methyl ribose (**12**) was facilitated with freshly prepared silver oxide (Ag_2_O) [34] and methyl iodide in *N*,*N*-dimethylformamide (DMF) with 95% yield, which is much milder than our former method (sodium hydride and methyl iodide in DMF with 88% yield). Next, the cleavage of the acetonide with acetic acid/acetic anhydride/H_2_SO_4_ afforded the key glycosylation donor (**7**) in 91% yield as a mixture of anomers (*α*:β = 2:3), which was used directly without further purification.

**Scheme 2:**
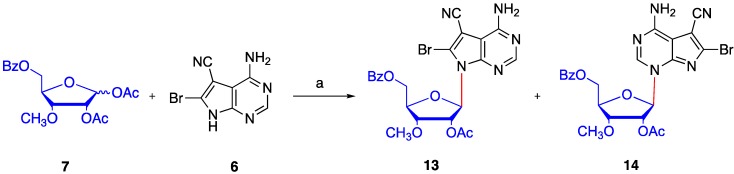
Vorbrüggen glycosylation of ribose (**7**) and nucleobase (**6**). Reagents and conditions: (**a**) BSA, TMSOTf, CH_3_CN, 80 °C, 4 h, 58%.

Then we started to investigate the crucial late-stage Vorbrüggen glycosylation [35,36,37] with nucleobase **6**, which was synthesized by our improved procedure from tetracyanoethylene in two steps [38]. In our previous total synthesis of mycalisine A, the yield of Vorbrüggen glycosylation was moderate (58% yield) [27]. Although the reaction proceeded smoothly to give only one spot of product in thin-layer chromatography (TLC), it was found that the *N*-9 glycosylation product **13** and the *N*-3 glycosylation product **14** were formed concurrently after purification (Scheme 2). The formation of regioisomers was also previously reported by us and several other groups [35,38,39,40]. During our total synthesis of naturally occurring 5′-deoxytoyocamycin and 5′-deoxysangivamycin, this dilemma was successfully solved by introducing a benzoyl group at *N*-6 of nucleobase **6**, which improved its solubility and reduced the pyrimidine ring’s electron density and nucleophilicity [38].

**Scheme 3:**
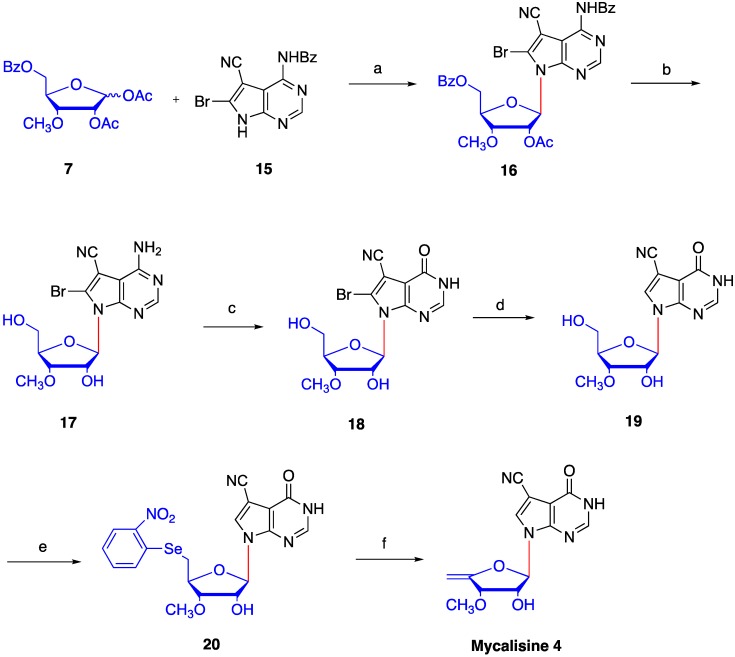
Total synthesis of mycalisine B. Reagents and conditions**:** (**a**) BSA, TMSOTf, CH_3_CN, 80 °C, 4h, 89%; (**b**) NH_3_/MeOH, 40 °C, 12 h, 87%; (**c**) NaNO_2_, HOAc, 60 °C, 8 h, 80% (**d**) Pd/C, H_2_, THF/MeOH, r.t., 4 h, 92%; (**e**) *O*-nitrophenylselenocyanate, Bu_3_P, Py, r.t., 4 h, 88%; (**f**) i. H_2_O_2_, THF, r.t., 2 h; ii. Et_3_N, Py, 50 °C, 5 h, 87%.

For this reason, we chose our newly developed nucleobase (**15**) to carry out the following synthesis (Scheme 3). Effective silylation of nucleobase with 3 equiv. of *N*,*O*-bis(trimethylsilyl)acetamide (BSA) in acetonitrile was first conducted. After the addition of ribose glycosylation donor (**7**), and followed by the addition of trimethylsilyl trifluoromethanesulfonate (TMSOTf), the reaction mixture was heated at 80 °C for 4 h. The corresponding nucleoside (**16**) was obtained in 89% yield without formation of the *N*-3 regioisomer. This reaction further demonstrated that 7-deazapurine nucleobase (**15**) could be used as a universal nucleobase for the synthesis of toyocamycin derivatives with the capacity to avoid the formation of *N*-3 nucleoside isomer. Next, global deprotection with saturated ammonia in methanol gave nucleoside (**17**) in 87% yield. Deamination with NaNO_2_ in acetic acid afforded 7-deazainosine (**18**) in 80% yields [41]. Then, debromination was performed by hydrogenation using 5% Pd/C as catalyst to give (**19**) in 92% yield [27]. It is noteworthy that we chose the synthetic sequence of debromination and subsequent deprotection during our total synthesis of mycalisine A. For purification reason, this change made the whole synthesis more convenient during our present work.

Subsequently, treatment nucleoside (**19**) with *O*-nitrophenylselenocyanate and tributyl phosphine in pyridine smoothly gave intermediate (**20**) in 88% yield [42]. Selenide (**20**) was then oxidized to the selenoxide intermediate with an excess of H_2_O_2_ in THF. Without further purification, the reaction mixture was directly treated with Et_3_N at 50 °C for 5 h. After removal of the solvent, purification of the residue afforded crystalline mycalisine B in 87% yield in two steps. It is noteworthy that mycalisine B was originally reported as an oil [7]. All its spectroscopic data are in accordance with those reported for mycalisine B [7] (See Supplementary Materials).

## 3. Conclusions

In summary, we have developed an expeditious and scalable approach for the total synthesis of mycalisine B in ten linear steps with 27.5% overall yield (based on the quantitative converting side product (**10**) to ribose (**9**)). The crucial ribose glycosylation donor (**7**) was synthesized from 1,2,3,5-tetra-*O*-acetylribose, which avoided oxidation and reduction reactions. Using *N*^4^-benzoyl-5-cyano-6-bromo-7*H*-pyrrolo[2,3-*d*]pyrimidine (**15**) as a nucleobase, the *N*-9 glycosylation product was obtained without the formation of *N*-3 isomer in Vorbrüggen glycosylation. We expected that the current total synthetic strategy can be widely used in the syntheses of mycalisine derivatives. However, this reported sequence continues to have some flaws, especially the double-bond formation reaction, which used the combination of *O*-nitrophenylselenocyanate and tributyl phosphine, which is expensive and will be further addressed in the future. Investigation of the biological activities of mycalisine B as potential antibiotics is ongoing, which will be reported in due course.

## 4. Materials and Methods

Unless otherwise specified, all the reagents were acquired from commercial sources and used directly. Acetonitrile, dichloromethane, dimethylformamide (DMF), and pyridine were all distilled from calcium hydride. NMR spectra (400 MHz/100 MHz) were recorded on an Advance DPX spectrometer (Bruker, Billerica, MA, USA) at room temperature with DMSO-*d*_6_ or CDCl_3_ as solvent. ^1^H NMR data are reported as the following format: chemical shift, multiplicity (s = singlet, d = doublet, t = triplet, br = broad, m = multiplet), coupling constant and integration. Specific rotations were acquired on an Autopol IV polarimeter (Rudolph, Hackettstown, NJ, USA). High resolution mass spectrometry (HRMS) data were measured with an AB Sciex TOF 4600 instrument (AB Sciex, Singapore). Melting points were measured on an X-4 digital melting point apparatus (Beijing Taike Corparation, Beijing, China). X-ray diffraction analysis was performed on a Bruker Smart Apex II system (Bruker, Billerica, MA, USA).

### 4.1. Synthesis of 1,2-O-isopropylidene-α-d-ribofuranose (9)

To a solution of 1,2,3,5-*O*-tetraacetyl-β-d-ribose (**8**) (200.0 g, 628 mmol) in acetone (1.5 L) iodine (9.6 g, 38 mmol) was added in portions at 0 °C under argon atmosphere. The obtained reaction mixture was stirred for 4 h at room temperature and then quenched with 500 mL saturated NaS_2_O_3_. The solvent was evaporated under reduced pressure and the residue was dissolved in CH_2_Cl_2_ (3 L). The solution was washed with distilled water (500 mL × 3), *sat.* NaHCO_3_ (500 mL × 3), brine (500 mL × 3), and dried with anhydrous Na_2_SO_4_. After filtration, the filtrate was evaporated under reduced pressure. Then the residue was dissolved in MeOH (1.5 L) and K_2_CO_3_ (10.0g, 72 mmol) was added. The mixture was stirred for 3 h at room temperature. After filtrated and evaporated to dryness under reduced pressure, the residue was recrystallized with PE (Petroleum ether)/EtOAc to give (**9**) as a white solid (104.1 g, 547 mmol, 87%). R*_f_*= 0.2 (CH_2_Cl_2_:MeOH = 15:1, *V:V*); m.p. 89−91 °C; ${\left[\alpha \right]}_{D}^{25}$ + 62.30 (*c* = 0.10, CH_3_OH); ^1^H NMR (400 MHz, DMSO-*d*_6_) *δ* 5.64 (d, *J* = 3.7 Hz, 1H), 4.97 (d, *J* = 6.7 Hz, 1H), 4.64 (t, *J* = 5.7 Hz, 1H), 4.43 (t, *J* = 3.9 Hz, 1H), 3.77 − 3.57 (m, 3H), 3.43–3.36 (m, 1H), 1.43 (s, 3H), 1.26 (s, 3H); ^13^C NMR (100 MHz, DMSO-*d*_6_) *δ* 111.7, 103.8, 80.8, 79.6, 71.0, 60.7, 27.1, 26.9; HRESIMS *m*/*z*: [M + Na]^+^ calcd. for C_8_H_14_O_5_, 213.0733; found, 213.0736.

### 4.2. Synthesis of 1,2-O-isopropylidene-5-O-benzoyl-α-d-ribofuranose (**11**)

To a solution of (**9**) (10.0 g, 52.57 mmol) in anhydrous CH_2_Cl_2_ (100 mL), pyridine (11.22 g, 141.96 mmol) was added. Then benzoyl chloride (11.09 g, 78.85 mmol) was slowly added to the solution at −10 °C. After addition, the reaction mixture was stirred for 8 h and quenched with ice water. The mixture was diluted with CH_2_Cl_2_ (500 mL) and washed with 5% HCl (150 mL × 2), *sat.* NaHCO_3_ (150 mL × 2), and brine (150 mL × 2). After drying over anhydrous MgSO_4_, the filtrate was evaporated to dryness under reduced pressure and purified by silica gel column to give a white solid (**11**) (9.58 g, 32.55 mmol, 62%) and (**10**) (7.34 g, 18.42 mmol, 35%).

Compound (**11**), R*_f_* = 0.15 (PE/EtOAc = 4:1, *V*/*V*); m.p. 86−87 °C; ${\left[\alpha \right]}_{D}^{25}$ + 30.36 (*c* = 0.112, CH_2_Cl_2_); ^1^H NMR (400 MHz, CDCl_3_) *δ* 8.05 (d, *J* = 7.4 Hz, 2H), 7.55 (t, *J* = 7.4 Hz, 1H), 7.42 (t, *J* = 7.7 Hz, 2H), 5.84 (d, *J* = 3.8 Hz, 1H), 4.69 (dd, *J* = 12.3, 2.2 Hz, 1H), 4.60 (t, *J* = 4.4 Hz, 1H), 4.44 (dd, *J* = 12.3, 5.3 Hz, 1H), 4.08 (ddd, *J* = 7.9, 5.2, 2.4 Hz, 1H), 3.94 (td, *J* = 9.7, 5.2 Hz, 1H), 2.61 (d, *J* = 10.3 Hz, 1H), 1.58 (s, 3H), 1.37 (s, 3H); ^13^C NMR (100 MHz, CDCl_3_) *δ* 166.6, 133.2, 129.9 (C × 3), 128.5 (C × 2), 112.9, 104.2, 78.6, 78.4, 72.3, 63.5, 26.6 (C × 2); HRESIMS *m*/*z*: [M + Na]^+^ calcd. for C_15_H_18_O_6_Na 317.0996, found 317.1102.

Compound (**10**), R*_f_* = 0.50 (PE/EtOAc = 4:1, *V*/*V*); m.p. 100−102 °C; ${\left[\alpha \right]}_{D}^{25}$ + 123.01 (*c* = 0.113, CH_2_Cl_2_); ^1^H NMR (400 MHz, CDCl_3_) *δ* 8.05 (dd, *J* = 15.1, 7.7 Hz, 4H), 7.64 − 7.51 (m, 2H), 7.42 (dt, *J* = 21.0, 7.7 Hz, 4H), 5.96 (d, *J* = 3.0 Hz, 1H), 5.09 − 4.99 (m, 2H), 4.71 (dd, *J* = 12.0, 3.3 Hz, 1H), 4.68 − 4.62 (m, 1H), 4.50 (dd, *J* = 12.0, 4.7 Hz, 1H), 1.60 (s, 3H), 1.36 (s, 3H); ^13^C NMR (100 MHz, CDCl_3_) *δ* 166.3, 165.8, 133.5, 133.2, 130.0 (C × 2), 129.8 (C × 2), 129.7, 129.2, 128.5 (C × 2), 128.4 (C × 2), 113.4, 104.6, 77.6, 75.8, 73.5, 63.3, 26.8 (C × 2); HRESIMS *m*/*z*: [M + Na]^+^ calcd. for C_22_H_22_O_7_Na 421.1258, found 421.1262.

### 4.3. Synthesis of 1,2-O-isopropylidene-3-O-methyl-5-O-benzoyl-α-d-ribofuranose (12)

To a solution of (**11**) (5.0 g, 16.98 mmol) in DMF (50 mL) Ag_2_O (9.84 g, 42.47 mmol) and CH_3_I (12.06 g, 84.94 mmol) were added at 0 °C under argon atmosphere. After addition, the reaction mixture was stirred for 5 h at room temperature and then filtered with celite. The filtrated was diluted with EtOAc (300 mL) and washed with distilled water (200 mL × 2), *sat.* NaHCO_3_ (200 mL × 2), brine (200 mL × 2), and dried with anhydrous Na_2_SO_4_. After filtration, the filtrate was evaporated under reduced pressure and purified by silica gel column to give a white solid (**12**) (4.98 g, 16.15 mmol, 95%). R*_f_* = 0.3 (PE/ EtOAc = 4:1, *V*/*V*); m.p. 74−75 °C; ${\left[\alpha \right]}_{D}^{25}$ + 72.82 (*c* = 0.103, CH_2_Cl_2_); ^1^H NMR (400 MHz, CDCl_3_) *δ* 8.05 (d, *J* = 7.6 Hz, 2H), 7.56 (t, *J* = 7.4 Hz, 1H), 7.43 (t, *J* = 7.7 Hz, 2H), 5.81 (d, *J* = 3.5 Hz, 1H), 4.71 (t, *J* = 3.9 Hz, 1H), 4.65 (dd, *J* = 12.2, 2.2 Hz, 1H), 4.41 (dd, *J* = 12.2, 5.0 Hz, 1H), 4.33 − 4.27 (m, 1H), 3.63 (dd, *J* = 9.1, 4.2 Hz, 1H), 3.49 (s, 3H), 1.61 (s, 3H), 1.37 (s, 3H); ^13^C NMR (100 MHz, CDCl_3_) *δ* 166.5, 133.3, 130.2, 130.0 (C × 2), 128.6 (C × 2), 113.4, 104.4, 81.3, 77.6, 76.7, 63.6, 58.7, 27.0, 26.7; HRESIMS *m*/*z*: [M + Na]^+^ calcd. for C_16_H_20_O_6_Na 331.1152, found 331.1136.

### 4.4. Synthesis of 1,2-O-diacetyl-3-O-methyl-5-O-benzoyl-d-ribofuranose (7)

To a solution of (**12**) (3.0 g, 9.73 mmol) in CH_3_CO_2_H (30 mL) and Ac_2_O (5.96 g, 58.38 mmol) concentrated H_2_SO_4_ (900 mg) was added dropwise in ice-bath conditions. After addition, the reaction mixture was stirred for 3h at room temperature. TLC detection showed the reaction was finished. The mixture was poured into H_2_O (100 mL) and extracted with CH_2_Cl_2_ (100 mL × 3). The combined organic layer was washed with distilled water (150 mL × 2), *sat.* NaHCO_3_ (150 mL × 2), brine (150 mL × 2), and dried with anhydrous Na_2_SO_4_. After filtration, the resulting solution was evaporated under reduced pressure and purified by silica gel column to give a viscous mass of monomers of (**7**) (3.12 g, 8.86 mmol, 91%, *α*:β = 2:3).

**7*β****:*R*_f_* = 0.25 (PE/EtOAc = 4:1, *V*/*V*); ${\left[\alpha \right]}_{D}^{25}$ − 11.96 (*c* = 0.092, CH_2_Cl_2_); ^1^H NMR (400 MHz, CDCl_3_) *δ* 8.07 (d, *J* = 7.4 Hz, 2H), 7.57 (t, *J* = 7.1 Hz, 1H), 7.44 (t, *J* = 7.5 Hz, 2H), 6.15 (s, 1H), 5.33 (d, *J* = 3.9 Hz, 1H), 4.65 (dd, *J* = 12.0, 2.8 Hz, 1H), 4.40 (dd, *J* = 12.1, 4.1 Hz, 1H), 4.35 − 4.28 (m, 1H), 4.07 (dd, *J* = 7.9, 4.2 Hz, 1H), 3.40 (s, 3H), 2.16 (s, 3H), 1.92 (s, 3H); ^13^C NMR (100 MHz, CDCl_3_) *δ* 169.8, 169.0, 166.2, 133.4, 130.0, 129.8 (C × 2), 128.5 (C × 2), 98.6, 79.9, 79.2, 73.2, 63.8, 59.4, 21.0, 20.8, HRESIMS *m*/*z*: [M + Na]^+^ calcd. for C_17_H_20_O_8_Na 375.1050, found 375.1047.

**7*α****:*R*_f_* = 0.20 (PE/ EtOAc = 4:1, *V*/*V*); ${\left[\alpha \right]}_{D}^{25}$ + 59.02 (*c* = 0.122, CH_2_Cl_2_); ^1^H NMR (400 MHz, CDCl_3_) *δ* 8.03 (d, *J* = 7.6 Hz, 2H), 7.57 (t, *J* = 7.4 Hz, 1H), 7.44 (t, *J* = 7.7 Hz, 2H), 6.41 (d, *J* = 4.6 Hz, 1H), 5.20 (dd, *J* = 6.3, 4.9 Hz, 1H), 4.51 (dd, *J* = 7.3, 4.9 Hz, 2H), 4.42 (dd, *J* = 12.8, 5.3 Hz, 1H), 3.96 (dd, *J* = 6.4, 3.9 Hz, 1H), 3.41 (s, 3H), 2.15 (s, 3H), 2.13 (s, 3H); ^13^C NMR (100 MHz, CDCl_3_) *δ* 170.0 (C × 2), 166.2, 133.4, 129.8 (C × 2), 129.6, 128.6 (C × 2), 94.5, 81.6, 78.2, 71.1, 64.3, 59.2, 21.2, 20.6; HRESIMS *m*/*z*: [M + Na]^+^ calcd. for C_17_H_20_O_8_Na 375.1050, found 375.1050.

### 4.5. Synthesis of N^4^-benzoyl-5-cyano-6-bromo-7-(2′-O-acetyl -3′-O-methyl-5′-O-benzoyl-β-d- ribofuranosyl) -7H-pyrrolo[2,3-d] pyrimidine (16)

To a suspended solution of *N^4^*-benzoyl-5-cyano-6-bromo-*7H*-pyrrolo[2,3-*d*] pyrimidine (**15**) (1.0 g, 2.92 mmol) in dry MeCN (15 mL), BSA (2.37 g, 11.68 mmol) was added and stirred for 20 min at 50 °C under argon atmosphere. After cooling to room temperature, the solution of (**7**) (2.06 g, 5.84 mmol) in dry MeCN (10 mL) along with TMSOTf (2.59 g, 11.68 mmol) were added to the reaction mixture at 0 °C. The mixture was stirred for 15 min before heating to 80 °C for 4h. Then the solution was poured into cold *sat.* NaHCO_3_ solution (30 mL) and extracted with EtOAc (100 mL × 2). The combined organic layer was washed with *sat.* NaHCO_3_ (30 mL × 3), brine (30 mL × 2), and dried with anhydrous Na_2_SO_4_, filtered and evaporated under reduced pressure. The residue was purified by a silica gel column (CH_2_Cl_2_: MeOH, *V*/*V* = 100:1) to afford (**16**) (1.65 g, 2.60 mmol, 89%) as white solid. R*_f_*= 0.40 (CH_2_Cl_2_/EtOAc = 5:1, *V*/*V*); m.p. 183−186 °C; ${\left[\alpha \right]}_{D}^{25}$ − 38.00 (*c* = 0.1, CH_3_OH); ^1^H NMR (400 MHz, DMSO-*d*_6_) *δ*: 11.55 (s, 1H), 8.65 (s, 1H), 8.05 (d, *J* = 7.6 Hz, 2H), 7.82 (d, *J* = 7.7 Hz, 2H), 7.65 (t, *J* = 7.2 Hz, 2H), 7.57 (t, *J* = 7.5 Hz, 2H), 7.46 (t, *J* = 7.7 Hz, 2H), 6.40~6.29 (m, 1H), 6.20 (d, *J* = 3.1 Hz, 1H), 4.80 (t, *J* = 6.4 Hz, 1H), 4.71 (dd, *J* = 12.3, 2.3 Hz, 1H), 4.56 − 4.45 (m, 1H), 4.44 − 4.34 (m, 1H), 3.46 (s, 3H), 2.13 (s, 3H); ^13^C NMR (100 MHz, DMSO-*d*_6_) *δ* 170.2, 167.4, 165.8 152.9, 151.8, 151.4, 133.9, 133.1, 129.6 (C × 2), 129.4 (C × 2), 129.1 (C × 2), 129.0 (C × 2), 128.9 (C × 2), 127.2, 114.2, 111.7, 91.3, 89.7, 80.1, 78.0, 72.6, 63.2, 59.1, 21.0; HRESIMS *m*/*z*: [M + Na]^+^ calcd. for C_29_H_24_BrN_5_O_7_Na 656.0751, found 656.0758.

### 4.6. Synthesis of 5-cyano-6-bromo-7-(3′-O-methyl-β-d-ribofuranosyl)-7H-pyrrolo[2,3-d] pyrimidine (17)

A solution of (**16**) (2.0 g, 3.15 mmol) in methanolic ammonia (saturated with NH_3_ at 0 °C for 2h, 20 mL) was placed in an autoclave and stirred at 40 °C for 12 h. Then the reaction mixture was concentrated to dryness and the residue was purified by a silica gel column (CH_2_Cl_2_: MeOH, *V*/*V* = 30:1) to afford (**17**) (1.05 g, 2.73 mmol, 87%) as white solid. R*_f_*= 0.45 (CH_2_Cl_2_:CH_3_OH = 20:1, *V*/*V*); m.p. 204−206 °C; ${\left[\alpha \right]}_{D}^{25}$ − 41.50 (*c* = 0.10, CH_3_OH); ^1^H NMR (400 MHz, DMSO-*d*_6_) *δ* 8.21 (s, 1H), 7.11 (s, 2H), 5.91 (d, *J* = 6.9 Hz, 1H), 5.76 (s, 1H), 5.55 (d, *J* = 6.6 Hz, 1H), 5.39 (dd, *J* = 7.8, 4.2 Hz, 1H), 5.25 (d, *J* = 6.0 Hz, 1H), 4.07 (d, *J* = 2.0 Hz, 1H), 3.92 (d, *J* = 3.0 Hz, 1H), 3.68 (dd, *J* = 10.2, 5.9 Hz, 1H), 3.63 − 3.50 (m, 1H), 3.44 (s, 3H); ^13^C NMR (100 MHz, DMSO-*d*_6_) *δ*: 156.1, 153.2, 150.0, 121.6, 114.1, 102.1, 90.9, 87.5, 84.1, 80.0, 70.7, 61.9, 57.7; HRESIMS *m*/*z*: [M + Na]^+^ calcd. for C_13_H_14_BrN_5_O_4_Na 406.0121, found 406.0126.

### 4.7. 4-carbonyl-5-cyano-6-bromo-7-(3′-O-methyl-β-d-ribofuranosyl)-7H-pyrrolo[2,3-d] pyrimidine (18)

To a solution of (**17**) (1.0 g, 2.6 mmol) in CH_3_CO_2_H (20 mL), an aqueous solution (NaNO_2_/H_2_O, 1.79 g/9.0 mL) was added slowly. After addition, the reaction mixture was heated to 60 °C for 8 h. After the reaction was finished (by TLC monitoring), the reaction mixture was concentrated to dryness. The residue was dissolved with CH_2_Cl_2_ (20 mL). After filtration, the resulting solution was evaporated under reduced pressure and the residue was purified by silica gel column to afford a white solid of (**18**) (0.795 g, 2.06 mmol, 80%). R*_f_*= 0.32 (CH_2_Cl_2_:CH_3_OH = 20:1, *V*/*V*); m.p. 219−220 °C; ${\left[\alpha \right]}_{D}^{25}$ − 67.50 (*c* = 0.08, CH_3_OH); ^1^H NMR (400 MHz, DMSO-*d*_6_) *δ*: 12.76 (brs, 1H), 8.14 (d, *J* = 3.6 Hz, 1H), 5.92 (d, *J* = 6.6 Hz, 1H), 5.58 (brs, 1H), 5.14 (s, 1H), 4.99 (brs, 1H), 4.02 − 3.99 (m, 1H), 3.91 (dd, *J* = 5.2, 3.3 Hz, 1H), 3.65 (dd, *J* = 11.7, 5.2 Hz, 1H), 3.54 (dd, *J* = 11.5, 5.0 Hz, 1H), 3.42 (s, 3H); ^13^C NMR (100 MHz, DMSO-*d*_6_) *δ*: 156.0, 148.6, 147.0, 119.4, 114.0, 109.5, 91.4, 91.2, 84.3, 80.1, 71.4, 62.1, 58.2; HRESIMS *m*/*z*: [M + Na]^+^ calcd. for C_13_H_13_BrN_4_O_5_Na 406.9962, found 406.9967.

### 4.8. Synthesis of 4-carbonyl-5-cyano-7-(3′-O-methyl-β-d-ribofuranosyl)-7H-pyrrolo[2,3-d] pyrimidine (19)

To a solution of (**18**) (1.0 g, 2.6 mmol) in THF (10 mL) and MeOH (10 mL) was added 10% Pd/C (100 mg) and Et_3_N (0.1 mL). After stirring at room temperature for 5 h under H_2_ atmosphere, the mixture was filtered with celite and the filtrated was concentrated to dryness under reduced pressure and purified by a silica gel column to afford a white solid of (**19**) (0.74 g, 2.42 mmol, 92%). R*_f_*= 0.15 (CH_2_Cl_2_:CH_3_OH = 20:1, *V*/*V*); m.p. 94−96 °C; ${\left[\alpha \right]}_{D}^{25}$ − 38.0 (*c* = 0.10, CH_3_OH); ^1^H NMR (400 MHz, DMSO-*d*_6_) *δ* 12.55 (s, 1H), 8.36 (s, 1H), 8.10 (s, 1H), 6.01 (d, *J* = 5.3 Hz, 1H), 5.60 (d, *J* = 6.2 Hz, 1H), 5.22 (t, *J* = 5.1 Hz, 1H), 4.47 (dd, *J* = 10.7, 5.4 Hz, 1H), 4.02 (d, *J* = 3.7 Hz, 1H), 3.83 (t, *J* = 4.3 Hz, 1H), 3.66 (m, 1H), 3.56 (m, 1H), 3.38 (s, 3H); ^13^C NMR (100 MHz, DMSO-*d*_6_) *δ* 156.9, 148.0, 146.6, 130.5, 114.8, 107.6, 88.1, 86.7, 83.2, 79.3, 73.8, 61.1, 57.7; HRESIMS *m*/*z*: [M + Na]^+^ calcd. for C_13_H_14_N_4_O_5_Na 329.0856, found 329.0848.

### 4.9. Synthesis of 4-carbonyl-5-cyano-7-(3′-O-methyl-5′-O-(2-nitrophenyl) selanyl -β-d-ribofuranosyl)-7H- pyrrolo [2,3-d] pyrimidine (20)

To a solution of (**11**) (91.5 mg, 0.3 mmol) in anhydrous pyridine (2 mL), *o*-nitrophenyl selenocyanate (204 mg, 0.9 mmol) and tributyl phosphine (0.225 mL, 0.9 mmol) were added under argon atmosphere. The reaction mixture was stirred for 4 h at room temperature and concentrated to dryness under reduced pressure. The residue was purified by a silica gel column to afford a yellow solid of (**12**) (129.1 mg, 0.26 mmol, 88%). R*_f_*= 0.48 (CH_2_Cl_2_:CH_3_OH = 20:1, *V*/*V*); m.p. 187−191 °C; ${\left[\alpha \right]}_{D}^{25}$ − 40.0 (*c* = 0.10, CH_3_OH); ^1^H NMR (400 MHz, DMSO-*d*_6_) *δ* 12.55 (d, *J* = 3.1 Hz, 1H), 8.35 (s, 1H), 8.25 (dd, *J* = 8.3, 1.2 Hz, 1H), 8.09 (d, *J* = 3.8 Hz, 1H), 7.78 (d, *J* = 7.7 Hz, 1H), 7.70 − 7.57 (m, 1H), 7.45 (t, *J* = 7.3 Hz, 1H), 6.01 (d, *J* = 5.5 Hz, 1H), 5.70 (d, *J* = 6.1 Hz, 1H), 4.67 (dd, *J* = 10.6, 5.3 Hz, 1H), 4.22 (dd, *J* = 10.5, 6.6 Hz, 1H), 3.89 (t, *J* = 4.4 Hz, 1H), 3.44 (d, *J* = 6.7 Hz, 2H), 3.37 (s, 3H); ^13^C NMR (100 MHz, DMSO-*d*_6_) *δ* 156.7, 148.0, 146.5 (C × 2), 134.2, 131.3, 130.7, 130.0, 126.3, 126.2, 114.4, 107.5, 88.1, 87.0, 82.3, 80.5, 72.8, 57.6, 28.5; HRESIMS *m*/*z*: [M + H]^+^ calcd. for C_19_H_18_N_5_O_6_Se 492.0417, found 492.0428.

### 4.10. Synthesis of mycalisine B (4)

To a solution of (**20**) (100 mg, 0.20 mmol) in THF (2 mL), 30% H_2_O_2_ (0.172 mL, 2 mmol) was added at ice-bath. After addition, the reaction mixture was stirred for 2 h at room temperature and concentrated to dryness under reduced pressure. The residue was dissolved in anhydrous pyridine (4 mL) and Et_3_N (0.4 mL, 0.3 mmol). Then the mixture was heated to 50 °C for 5 h. After cooling, the reaction mixture was concentrated under reduced pressure. The obtained residue was purified by a silica gel column to afford a white solid of mycalisine B (**4**) (49.7 mg, 0.17 mmol, 87%). R*_f_*= 0.32 (CH_2_Cl_2_/CH_3_OH = 20:1, *V*/*V*); m.p. 85−88 °C; ${\left[\alpha \right]}_{D}^{25}$ − 70.0 (*c* = 0.10, CH_3_OH); ^1^H NMR (400 MHz, DMSO-*d*_6_) *δ* 12.26 (brs, 1H), 8.41 (s, 1H), 8.12 (s, 1H), 6.25 (d, *J* = 7.0 Hz, 1H), 5.83 (s, 1H), 4.83 (m, 1H), 4.45 (d, *J* = 1.5 Hz, 1H), 4.32 (d, *J* = 1.6 Hz, 1H), 4.21 (d, *J* = 4.7 Hz, 1H), 3.39 (s, 3H); ^13^C NMR (100 MHz, DMSO-*d*_6_) *δ* 157.4, 156.6, 148.5, 146.8, 130.9, 114.2, 107.8, 87.8, 87.6, 87.4, 78.4, 72.4, 56.2; HRESIMS *m*/*z*: [M + H]^+^ calcd. for C_13_H_13_N_4_O_4_ 289.0931, found 289.0937.

## Supplementary Materials

The NMR spectra of compound Mycalisine **4**, **7**–**12**, **16**–**20** and crystallographic data of compound **11** are available online at https://www.mdpi.com/1660-3397/17/4/226/s1.
