# Supplementary material for: Total Synthesis of Mycalisine B
Source: Mar Drugs. 2019 Apr 14;17(4):226. doi: 10.3390/md17040226 (PMC6520845; doi:10.3390/md17040226)
Supplement: Supplementary file 1 [file marinedrugs-17-00226-s001.pdf]

Total Synthesis of Mycalisine B

Haixin Ding, Zhizhong Ruan, Peihao Kou, Xiangyou Dong, Jiang Bai, Qiang Xiao *

Key Laboratory of Organic Chemistry in Jiangxi Province, Institute of Organic Chemistry, Jiangxi Science & Technology Normal University, Nanchang 330013, China; dinghaixin_2010@163.com (H.D.); ruanzhizhong0404@yeah.net (R.Z); koupeihao92@yeah.net (B.K.); dongxiangyou93@yeah.net (X.D.); mtbaijiang@yeah.net (J.B.); xiaoqiang@tsinghua.org.cn (Q.X.).

* Correspondence: xiaoqiang@tsinghua.org.cn; Tel.: +86-791-86422903 (Q.X.).

Supplementary data index

Table 1. Comparison NMR data of Mycalisine B between literature values and test values S2
Fig. 1. ^{1}H NMR and ^{13}C NMR of Mycalisine B S3
Fig. 2. ^{1}H NMR and ^{13}C NMR of compound **7** S5
Fig. 3. ^{1}H NMR and ^{13}C NMR of compound **9** S9
Fig. 4. ^{1}H NMR and ^{13}C NMR of compound **10** S11
Fig. 5. ^{1}H NMR and ^{13}C NMR of compound **11** S13
Fig. 6. ^{1}H NMR and ^{13}C NMR of compound **12** S15
Fig. 7. ^{1}H NMR and ^{13}C NMR of compound **16** S17
Fig. 8. ^{1}H NMR and ^{13}C NMR of compound **17** S19
Fig. 9. ^{1}H NMR and ^{13}C NMR of compound **19** S21
Fig. 10. ^{1}H NMR and ^{13}C NMR of compound **20** S23
Crystallographic data of compound **11** S25

Table 1. Comparison NMR data of Mycalisine B between literature values and test values

Pos.	Mycalisine B (in DMSO-d_6)			
	δ_C (lit.)	δ_C (test)	δ_H (lit.)	δ_H (test)
2	147.2	146.8		
4	157.5	157.4		
4a	108.4	107.8		
5	88.5	87.8		
6	131.3	130.9		
7a	149.0	148.5		
1′	88.2	87.6	6.30(d, 7.1 Hz, 1H)	6.5(d, 7.0 Hz, 1H)
2′	72.1	72.4	4.84(dd, 7.1, 4.9 Hz, 1H)	4.83(m, 1 H)
3′	78.9	78.4	4.24(d, 4.9 Hz, 1H)	4.21(d, 4.7 Hz, 1H)
4′	157.5	156.6		
5′	88.0	87.4	4.52(d, 2.2Hz, 1H);<br>4.39(d, 2.2Hz, 1H)	4.45(d, 1.5 Hz, 1H);<br>4.39(d, 1.6 Hz, 1H)
CN	114.8	114.2		
OMe	56.6	56.2	3.43 (s, 3H)	3.39(s, 3H)

Fig.1. ^{1}H NMR and ^{13}C NMR of Mycalisne B

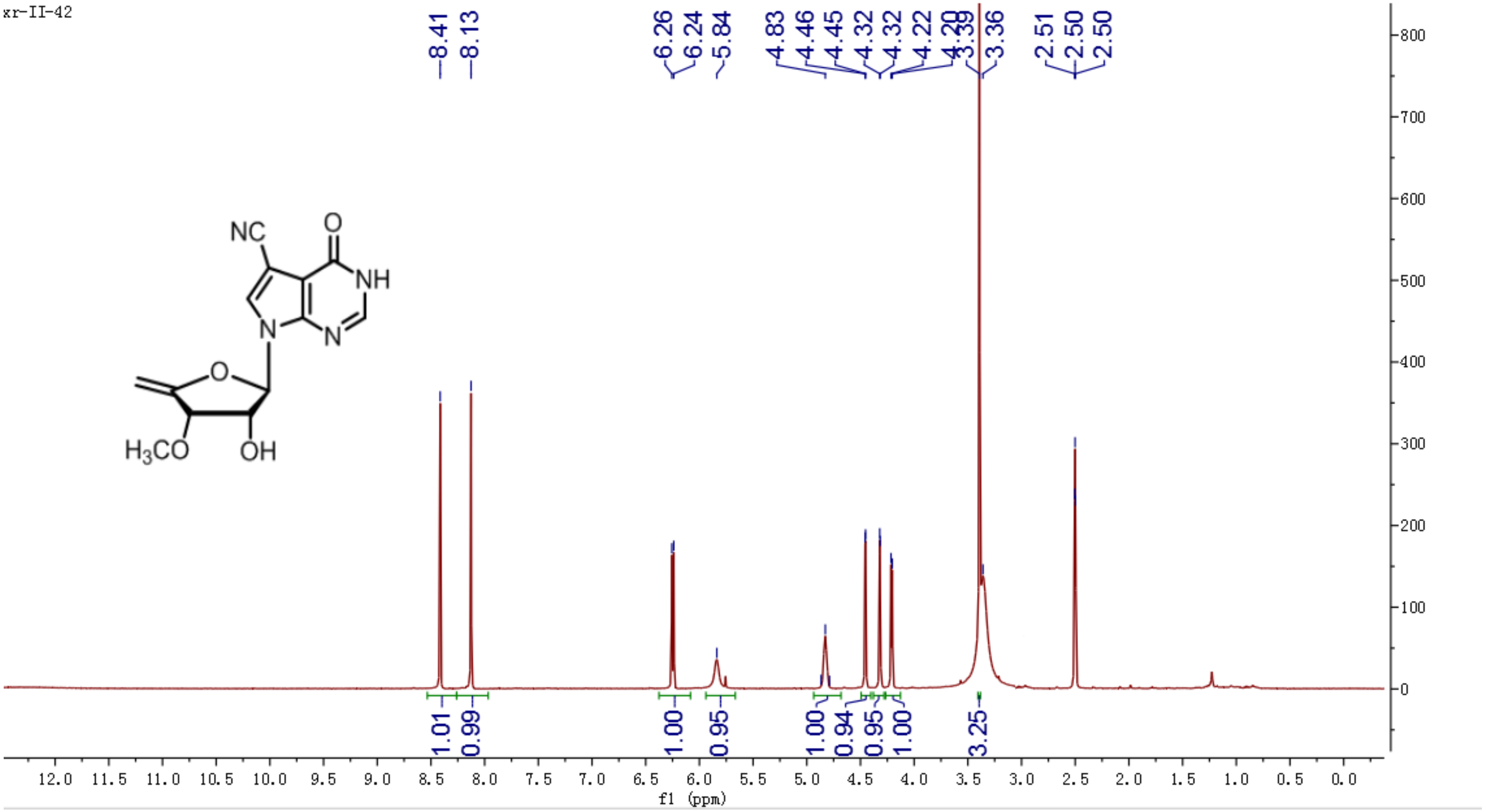

Fig.1. ^{1}H NMR and ^{13}C NMR of Mycalisne B

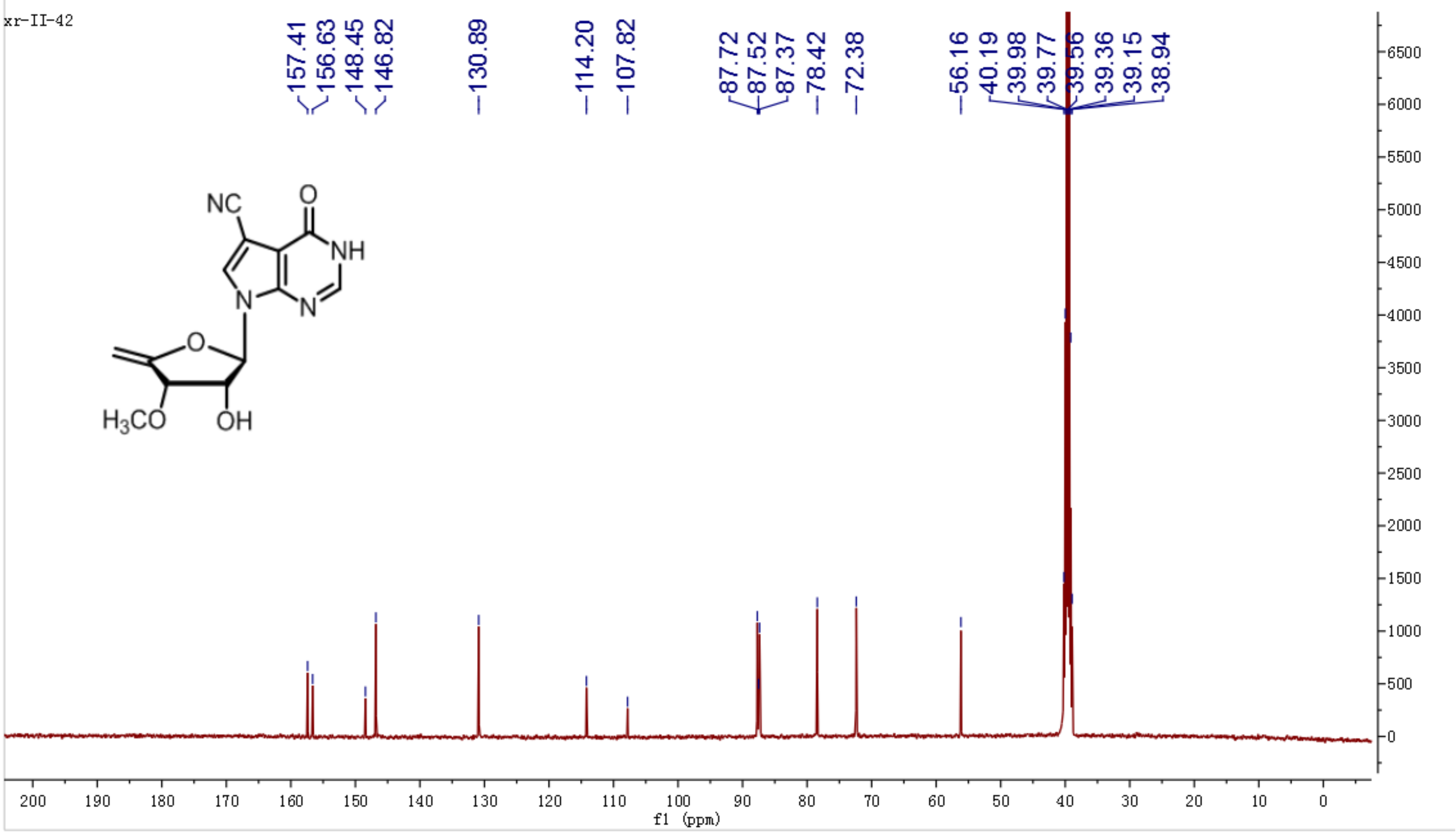

Fig. 2. ^{1}H NMR and ^{13}C NMR of compound **7β**

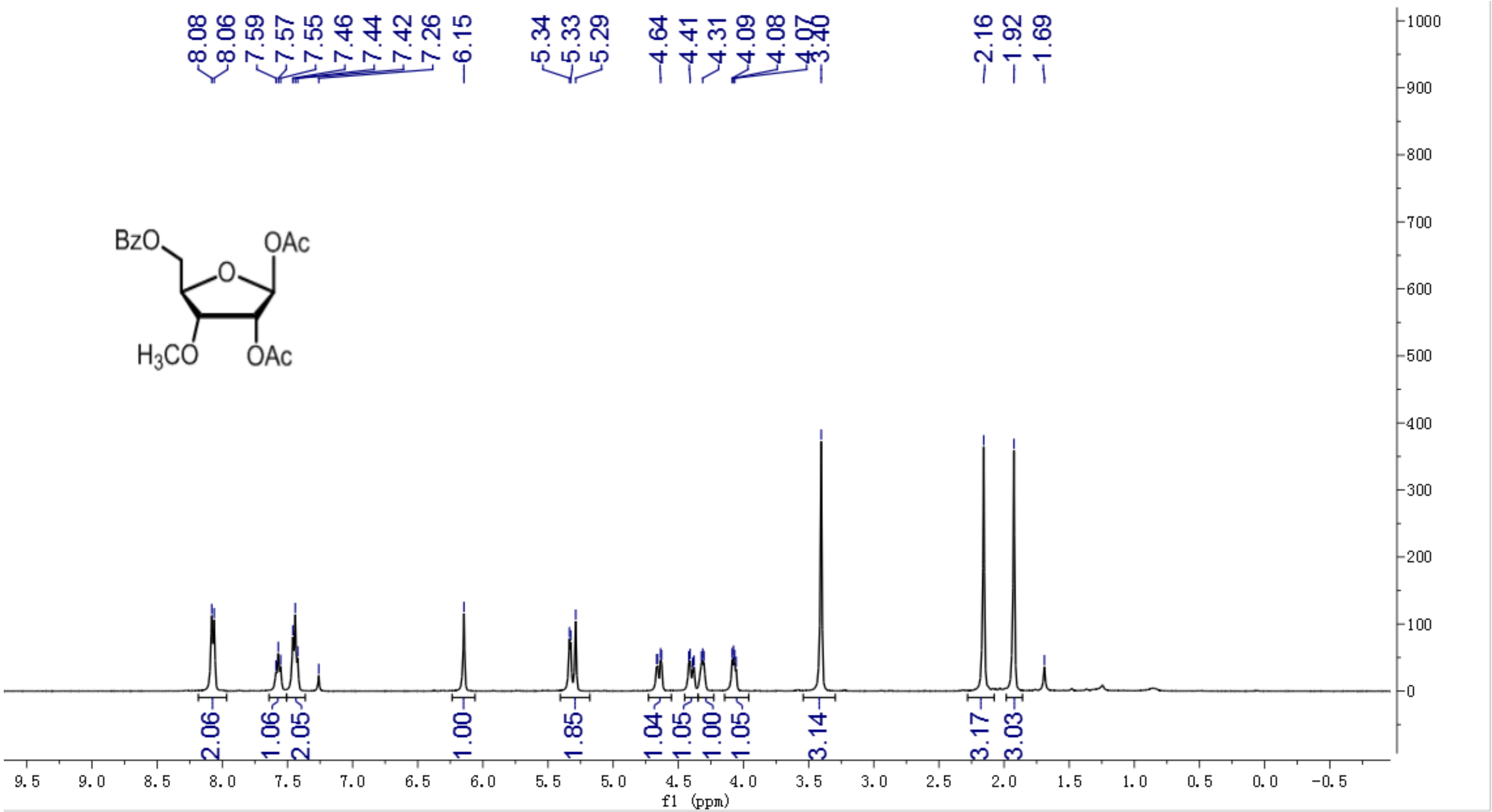

Fig. 2. ^{1}H NMR and ^{13}C NMR of compound **7β**

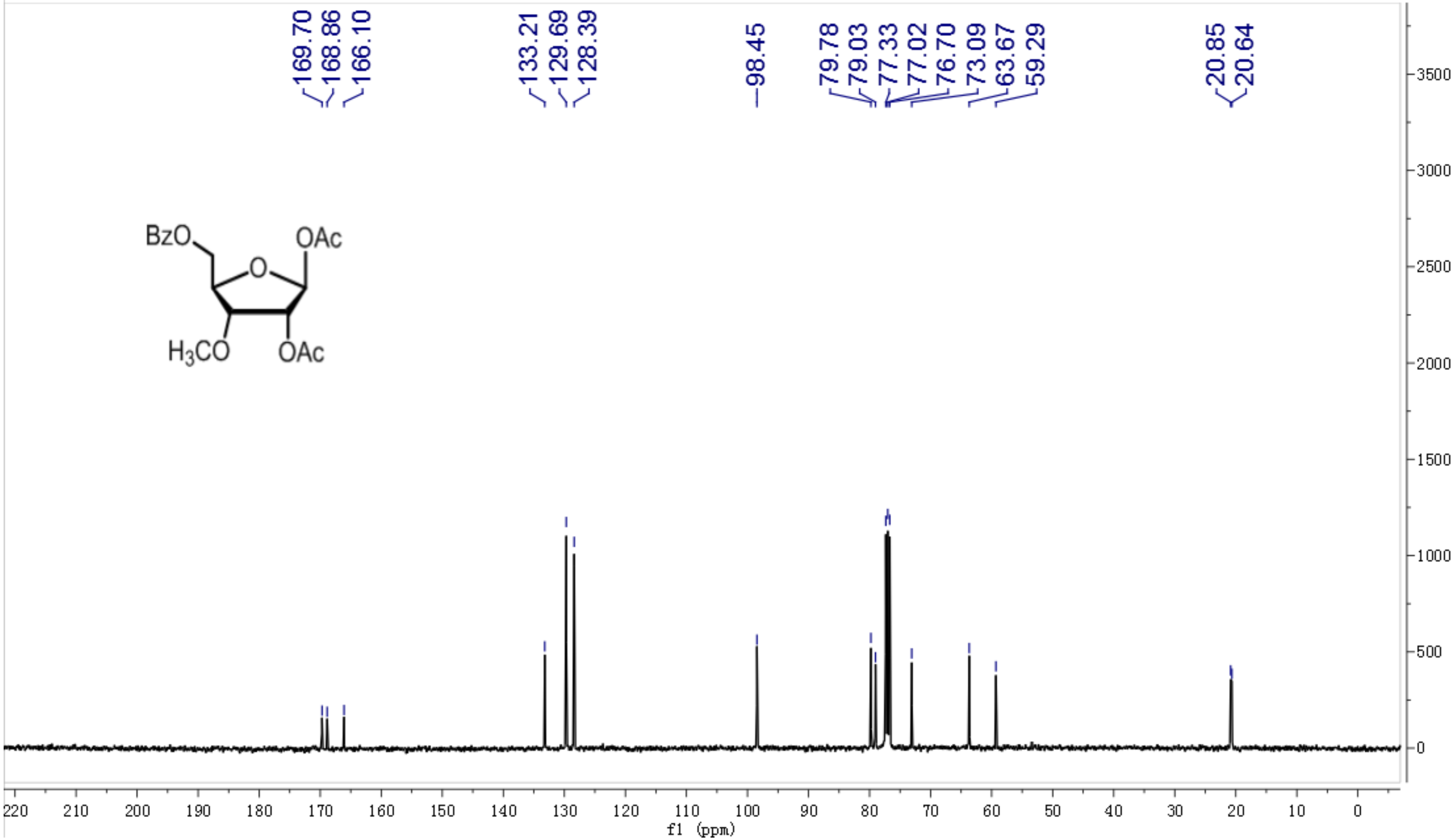

Fig. 2. ^{1}H NMR and ^{13}C NMR of compound **7α**

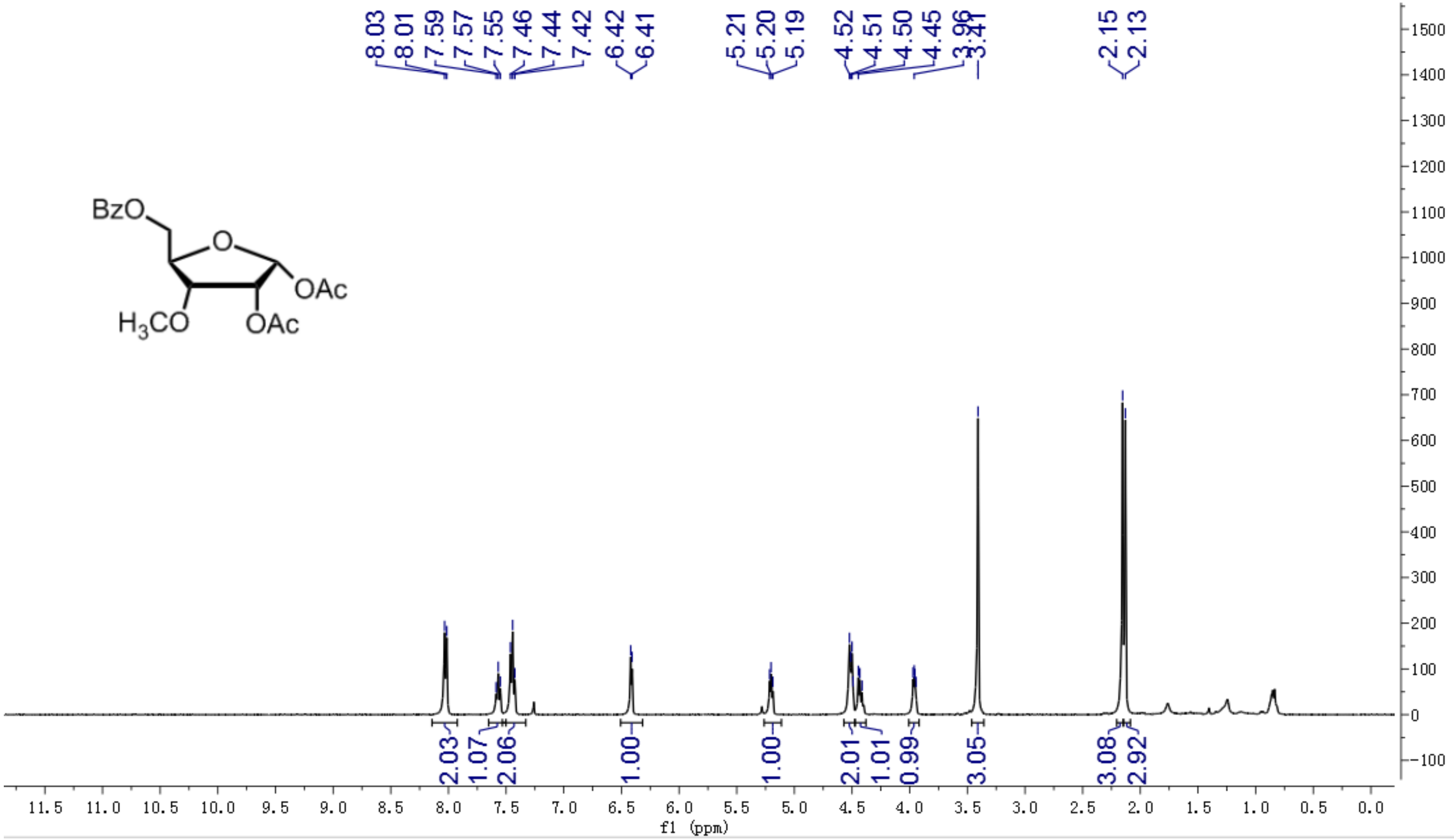

Fig. 2. ^{1}H NMR and ^{13}C NMR of compound **7α**

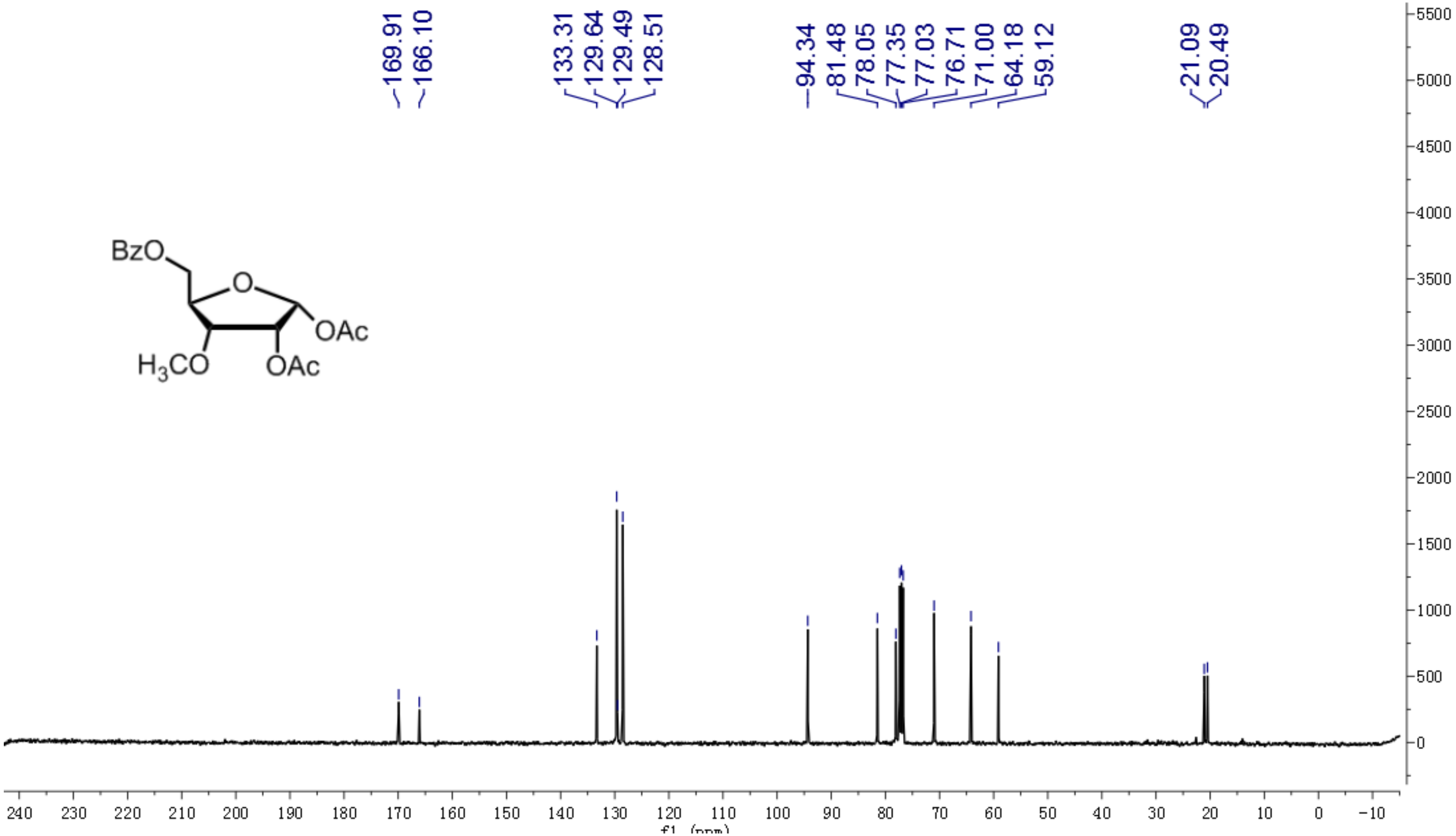

Fig. 3. ^{1}H NMR and ^{13}C NMR of compound **9**

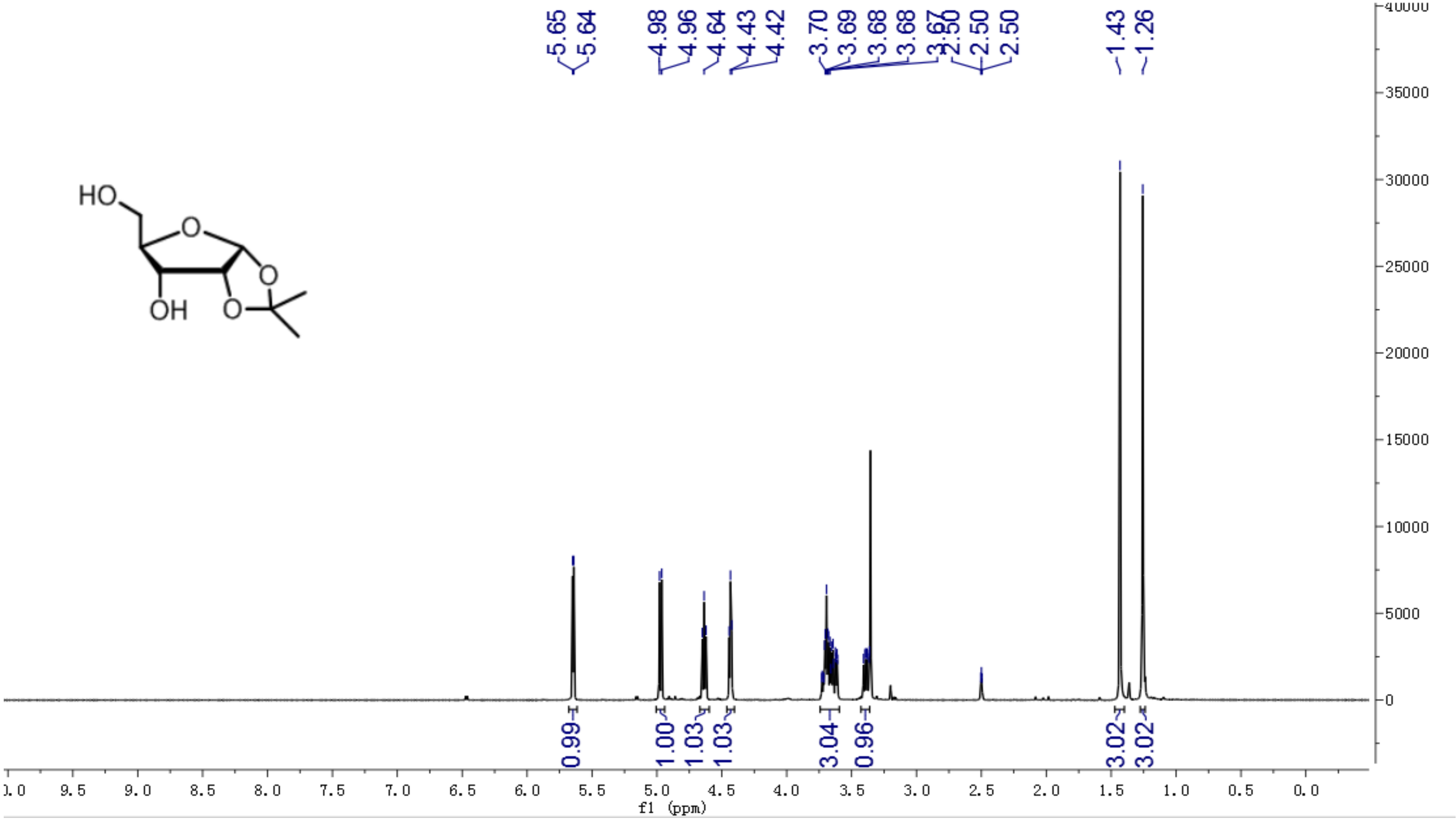

Fig. 3. ^{1}H NMR and ^{13}C NMR of compound **9**

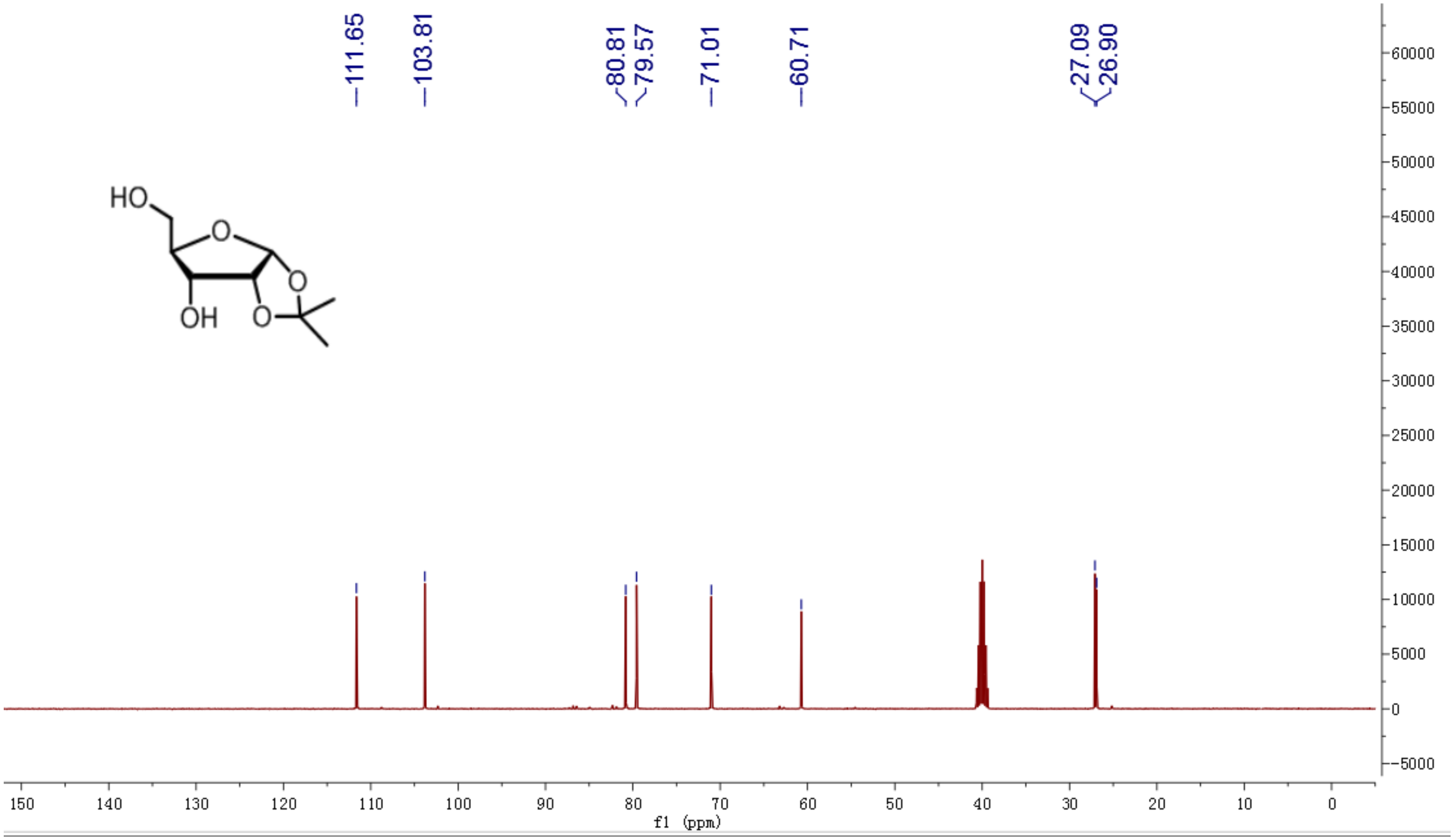

Fig. 4. ^{1}H NMR and ^{13}C NMR of compound **10**

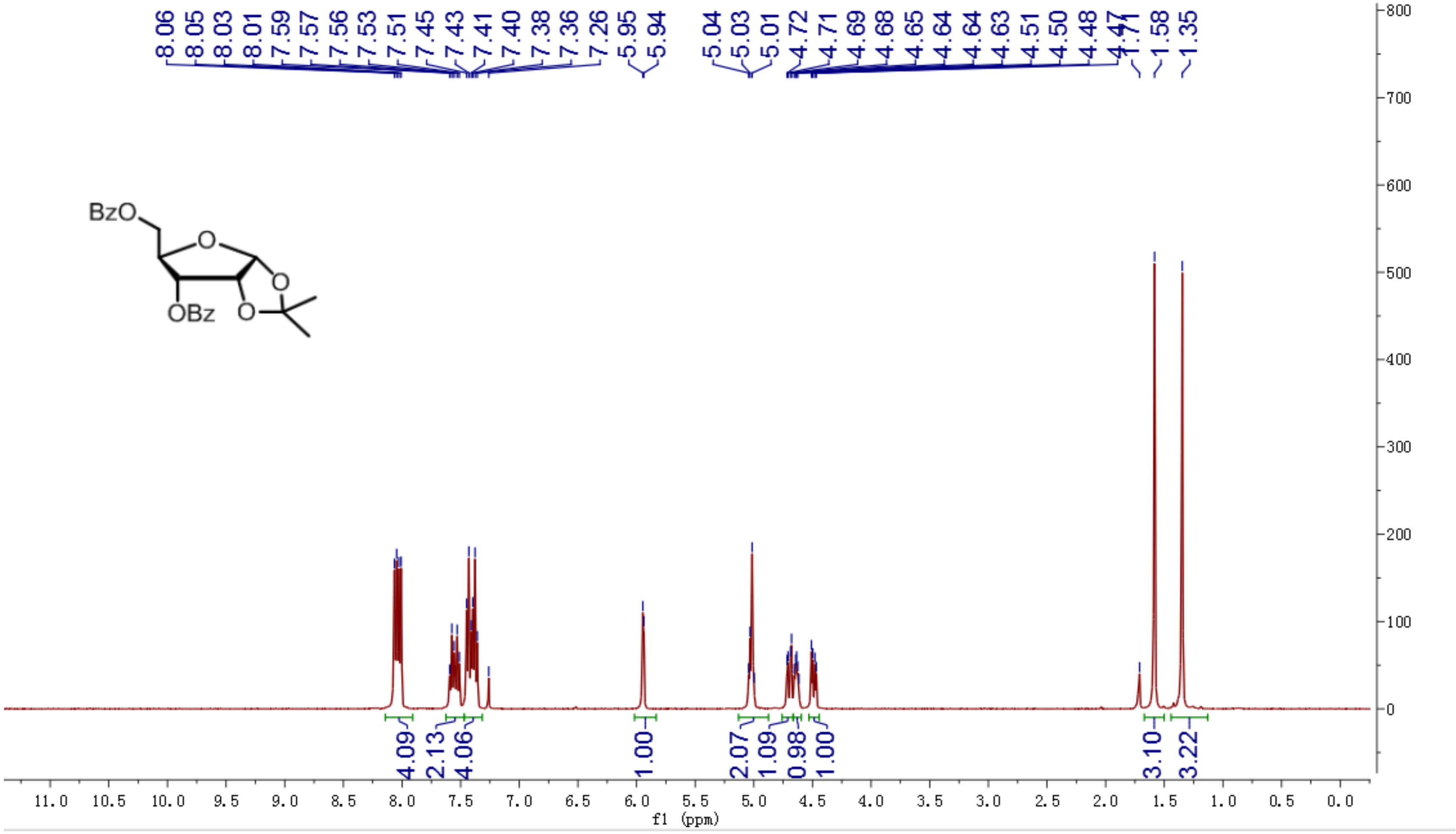

Fig. 4. ^{1}H NMR and ^{13}C NMR of compound **10**

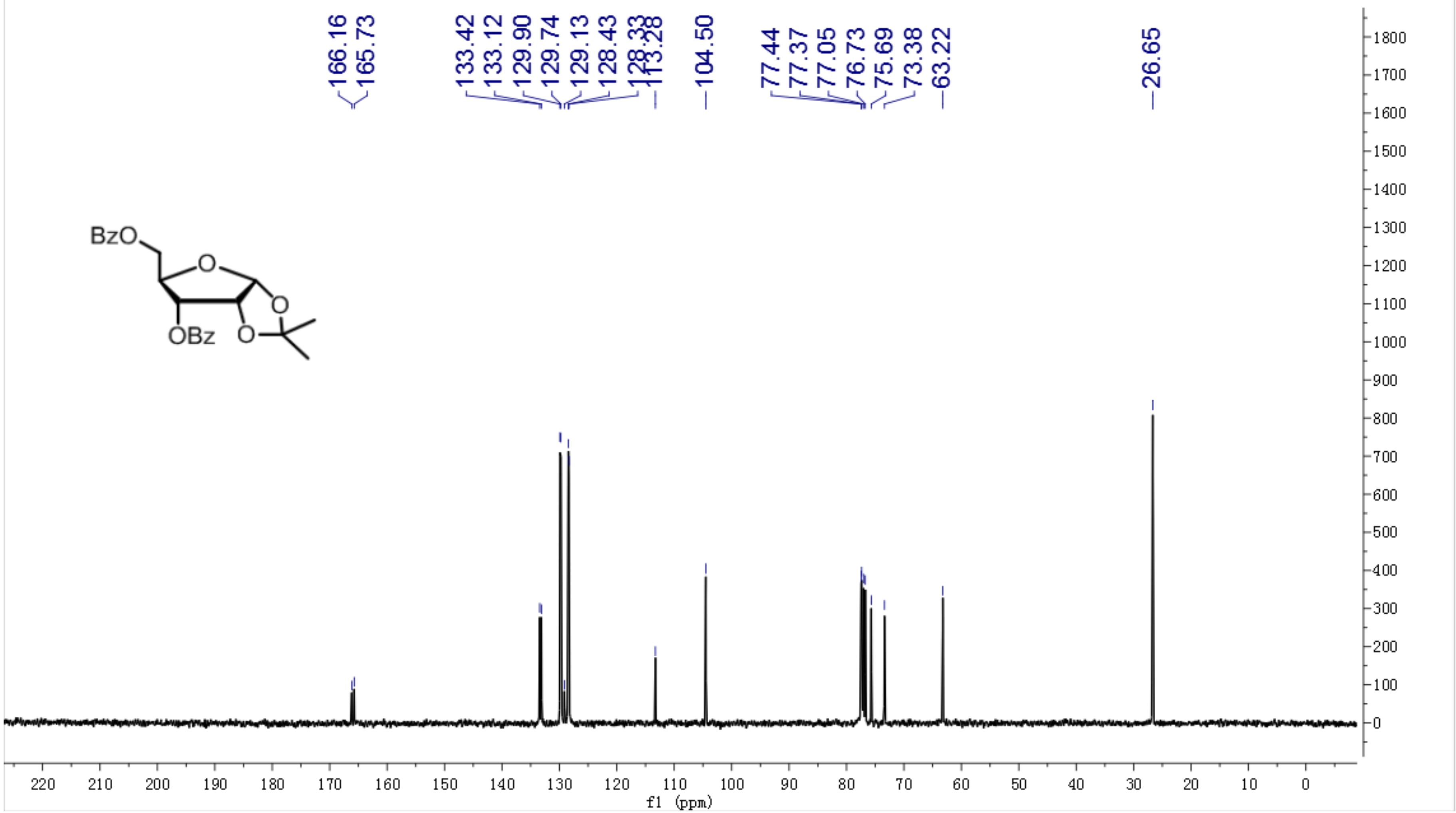

Fig.5. ^{1}H NMR and ^{13}C NMR of compound **11**

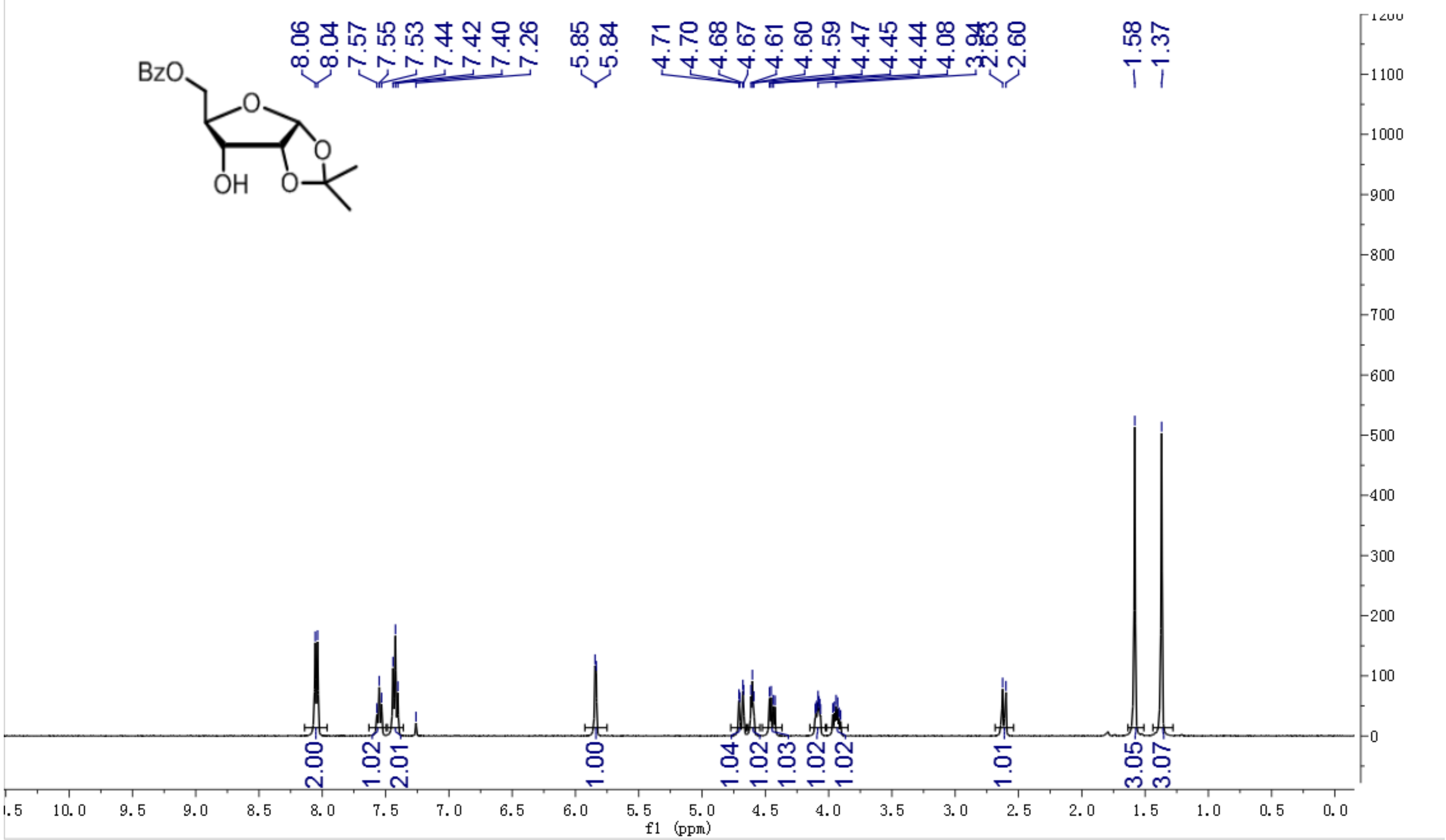

Fig.5. ^{1}H NMR and ^{13}C NMR of compound **11**

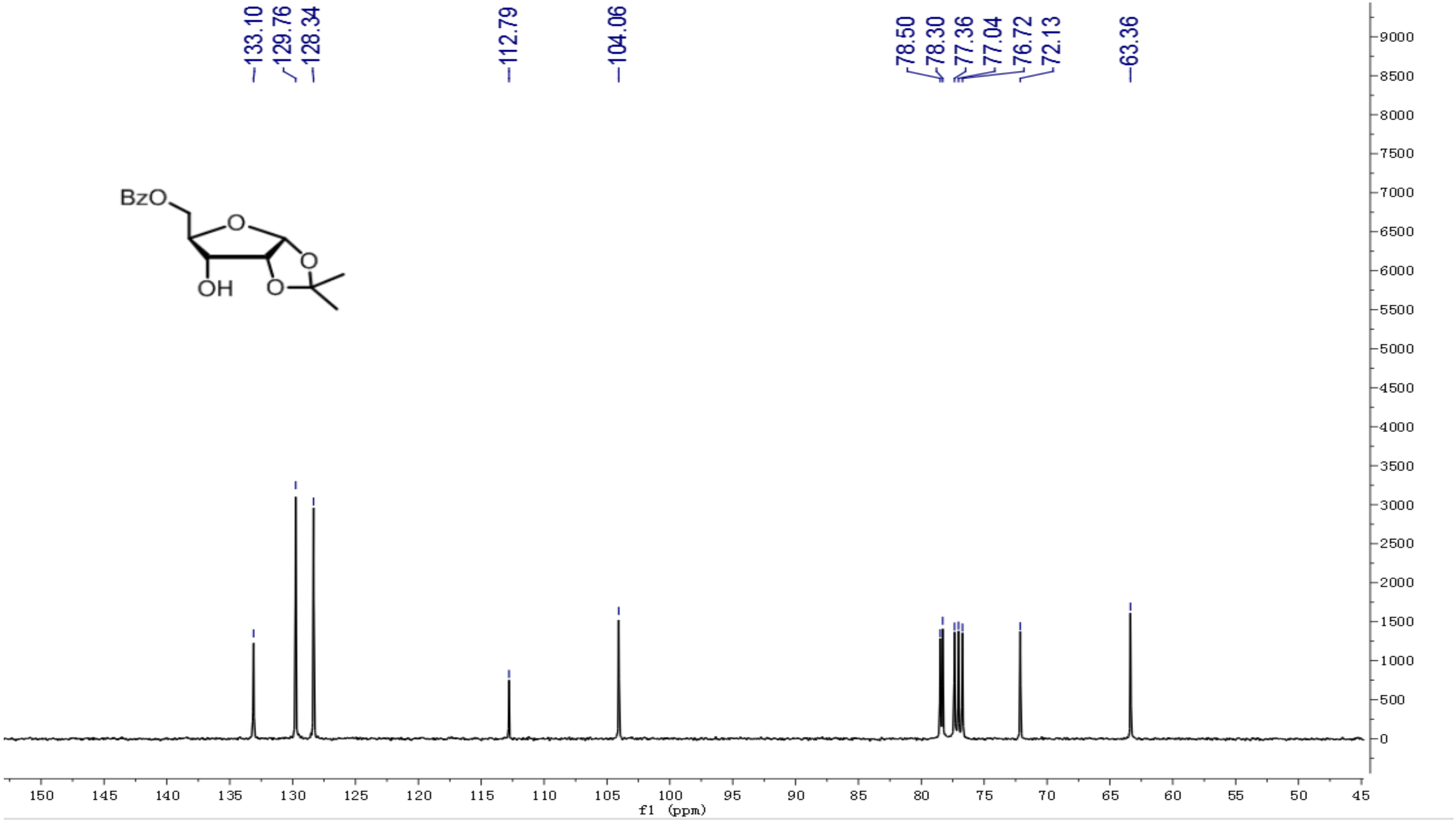

Fig.6. ^{1}H NMR and ^{13}C NMR of compound **12**

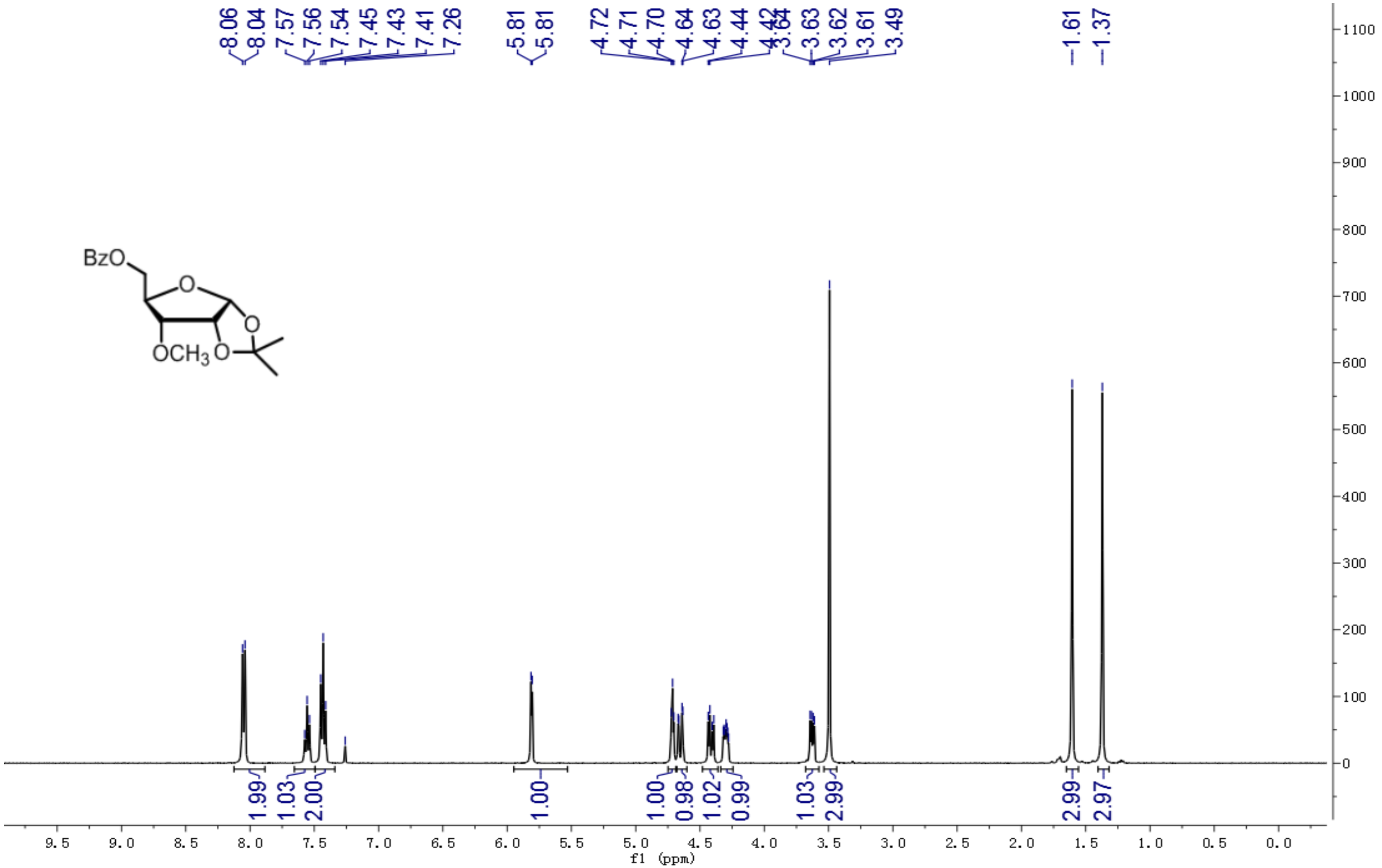

Fig.6. ^{1}H NMR and ^{13}C NMR of compound **12**

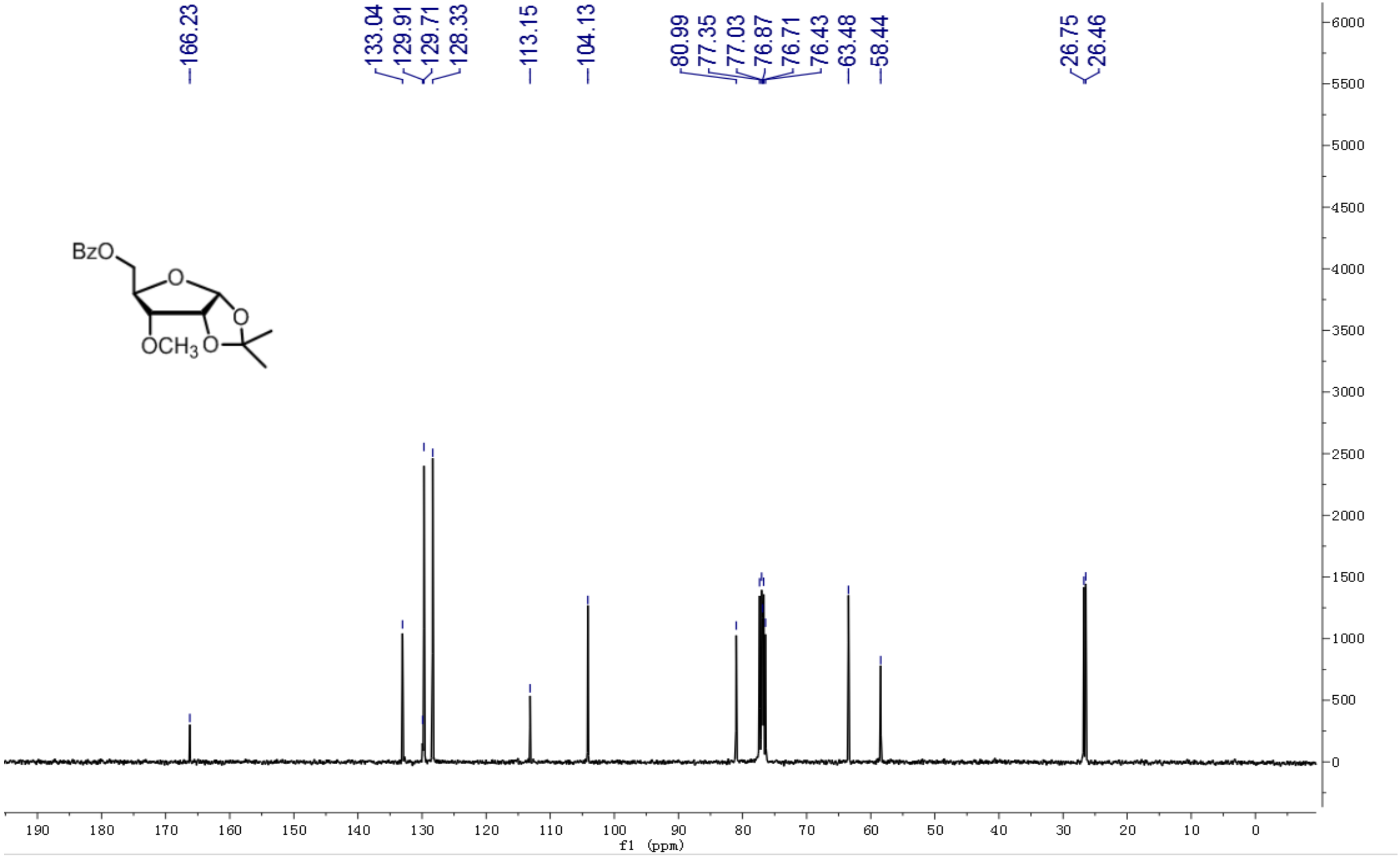

Fig.7. ^{1}H NMR and ^{13}C NMR of compound **16**

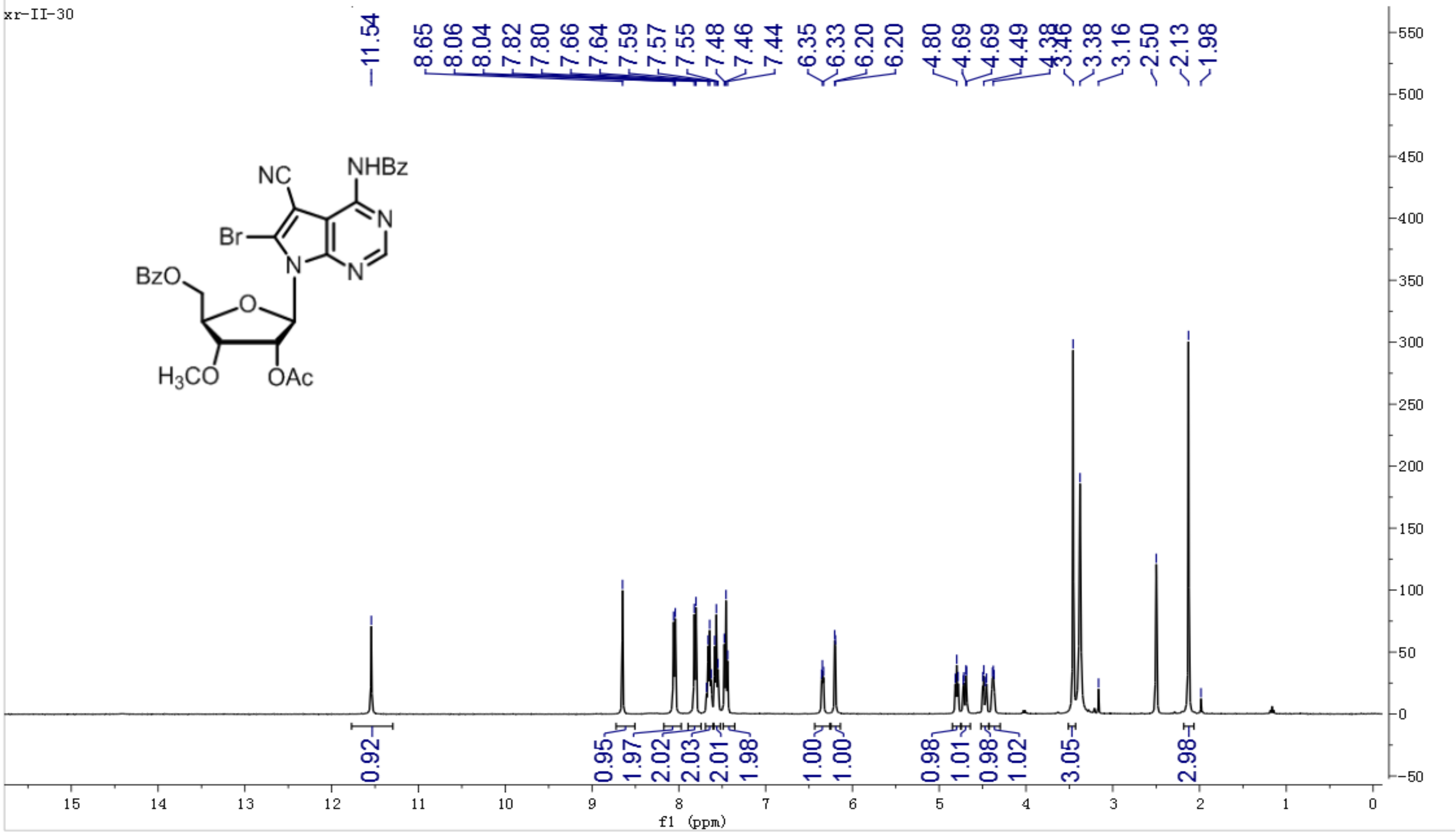

Fig.7. ^{1}H NMR and ^{13}C NMR of compound **16**

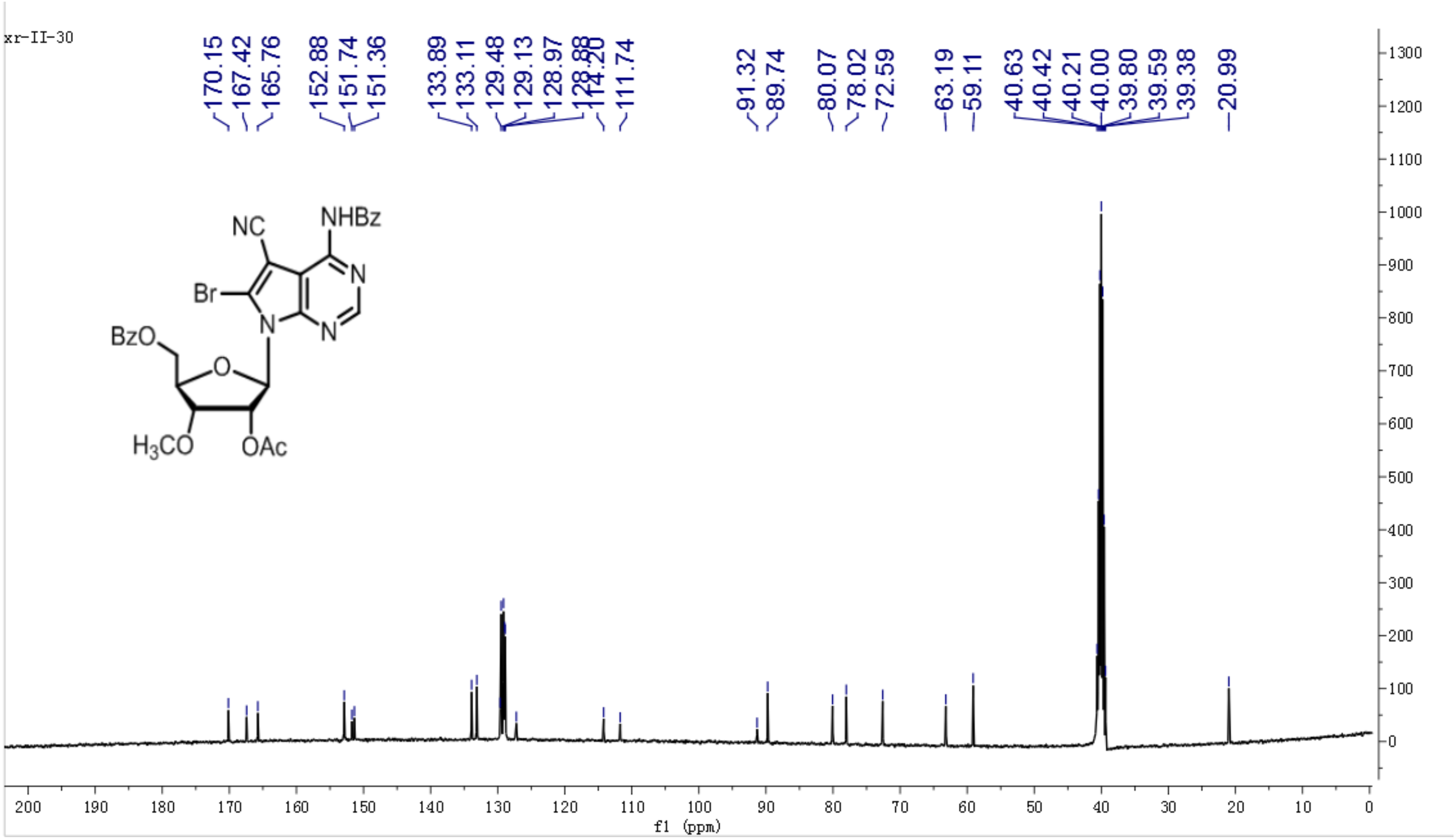

Fig.8. ^{1}H NMR and ^{13}C NMR of compound **17**

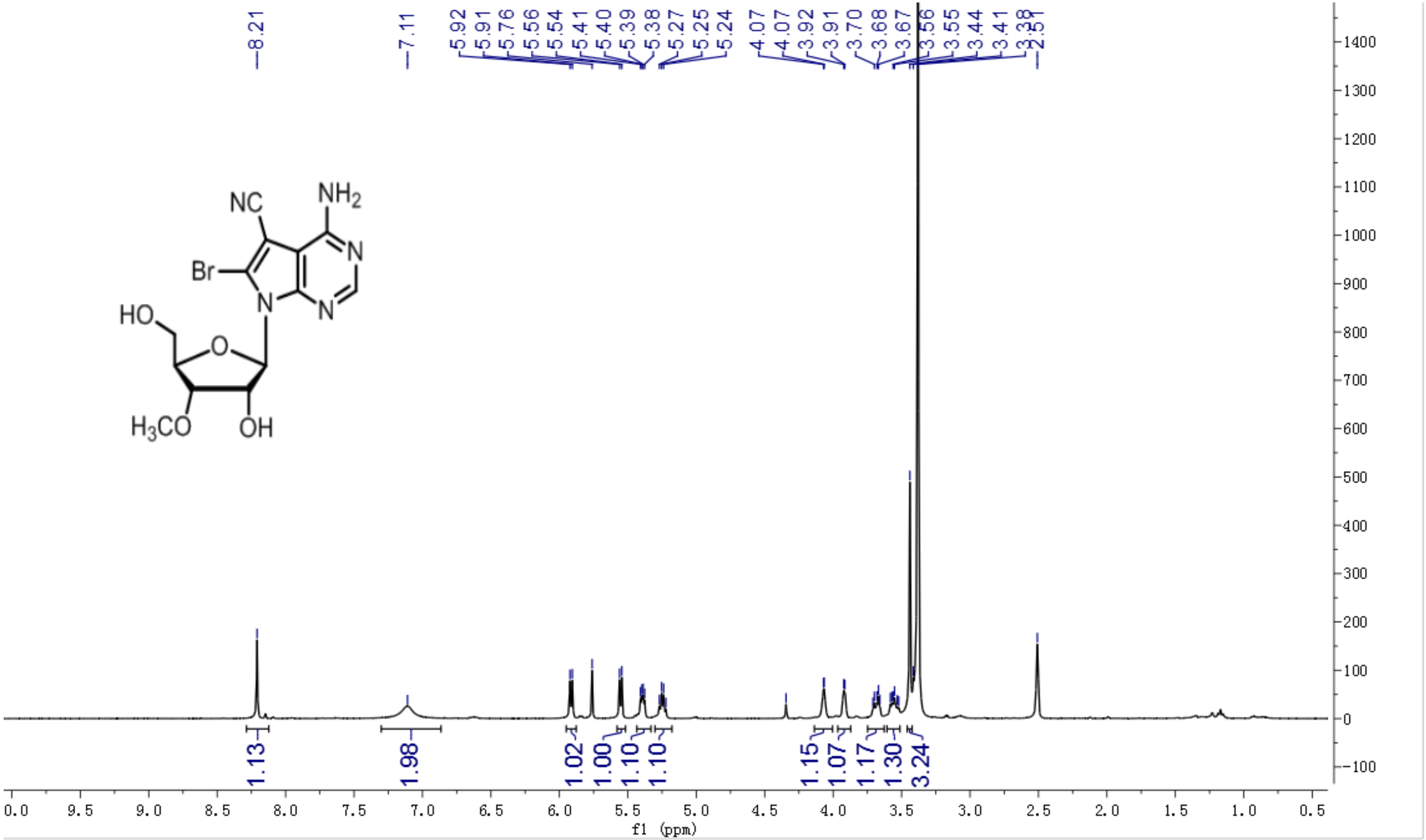

Fig.8. ^{1}H NMR and ^{13}C NMR of compound **17**

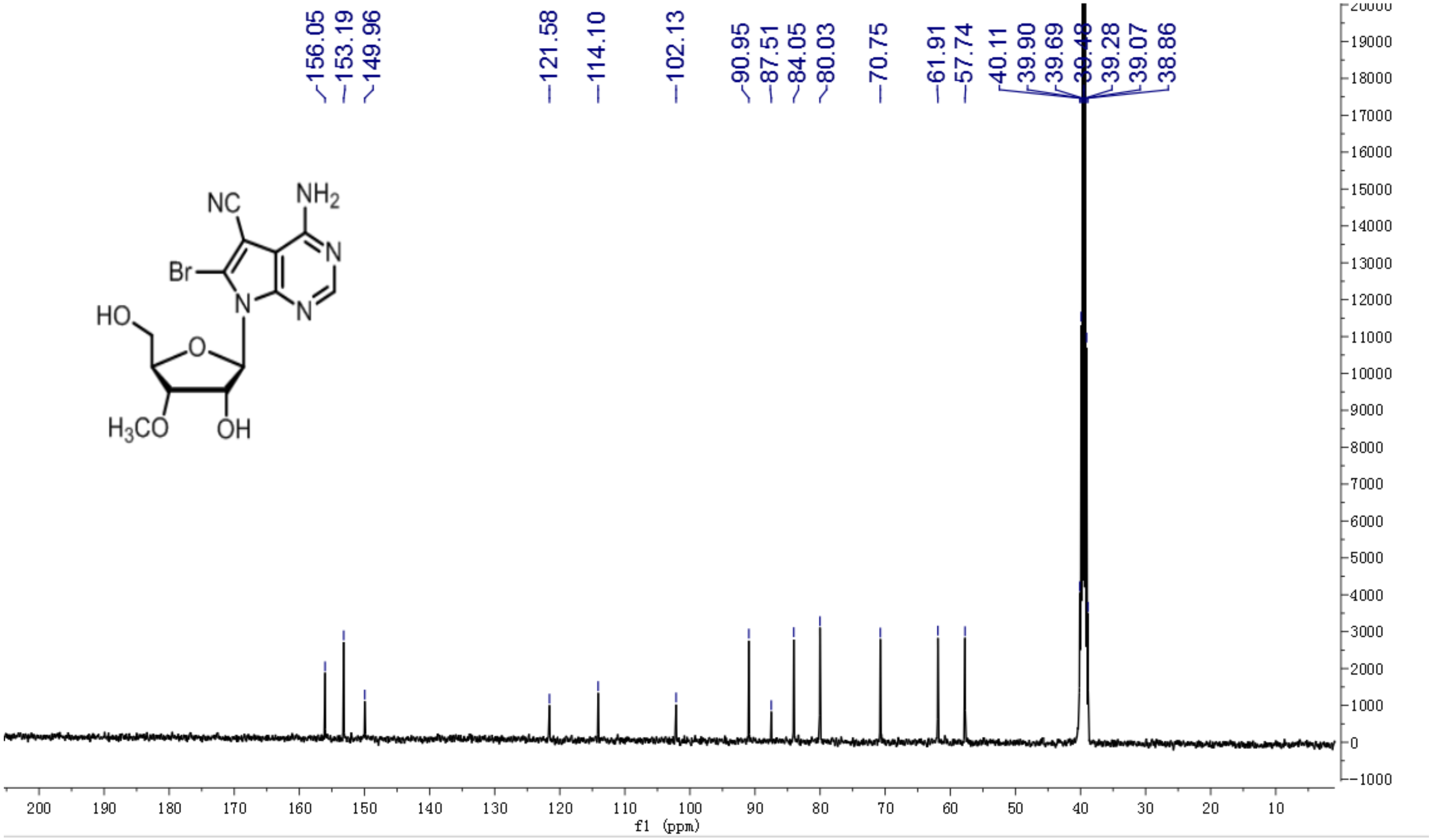

Fig.9. ^{1}H NMR and ^{13}C NMR of compound **19**

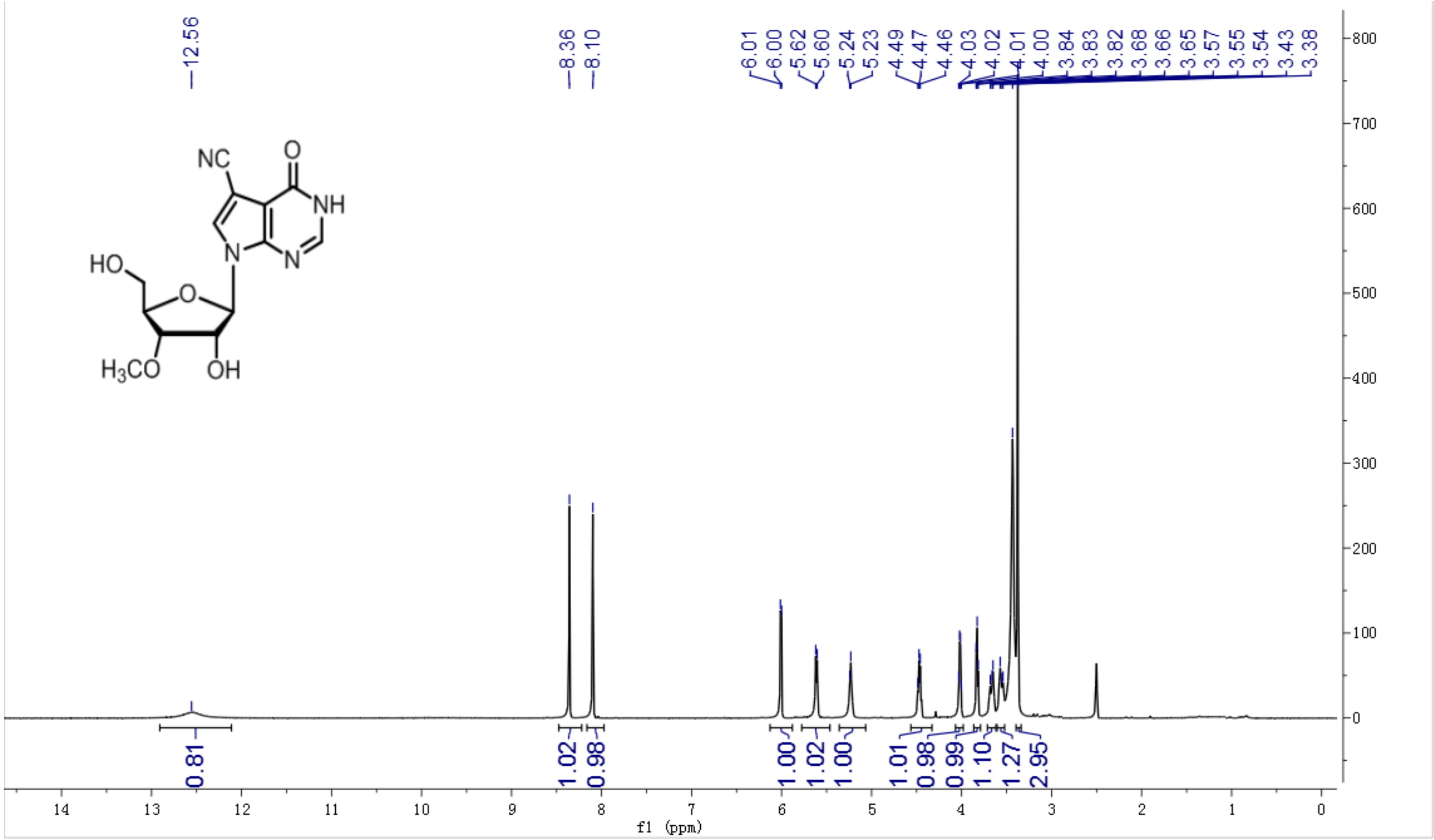

Fig.9. ^{1}H NMR and ^{13}C NMR of compound **19**

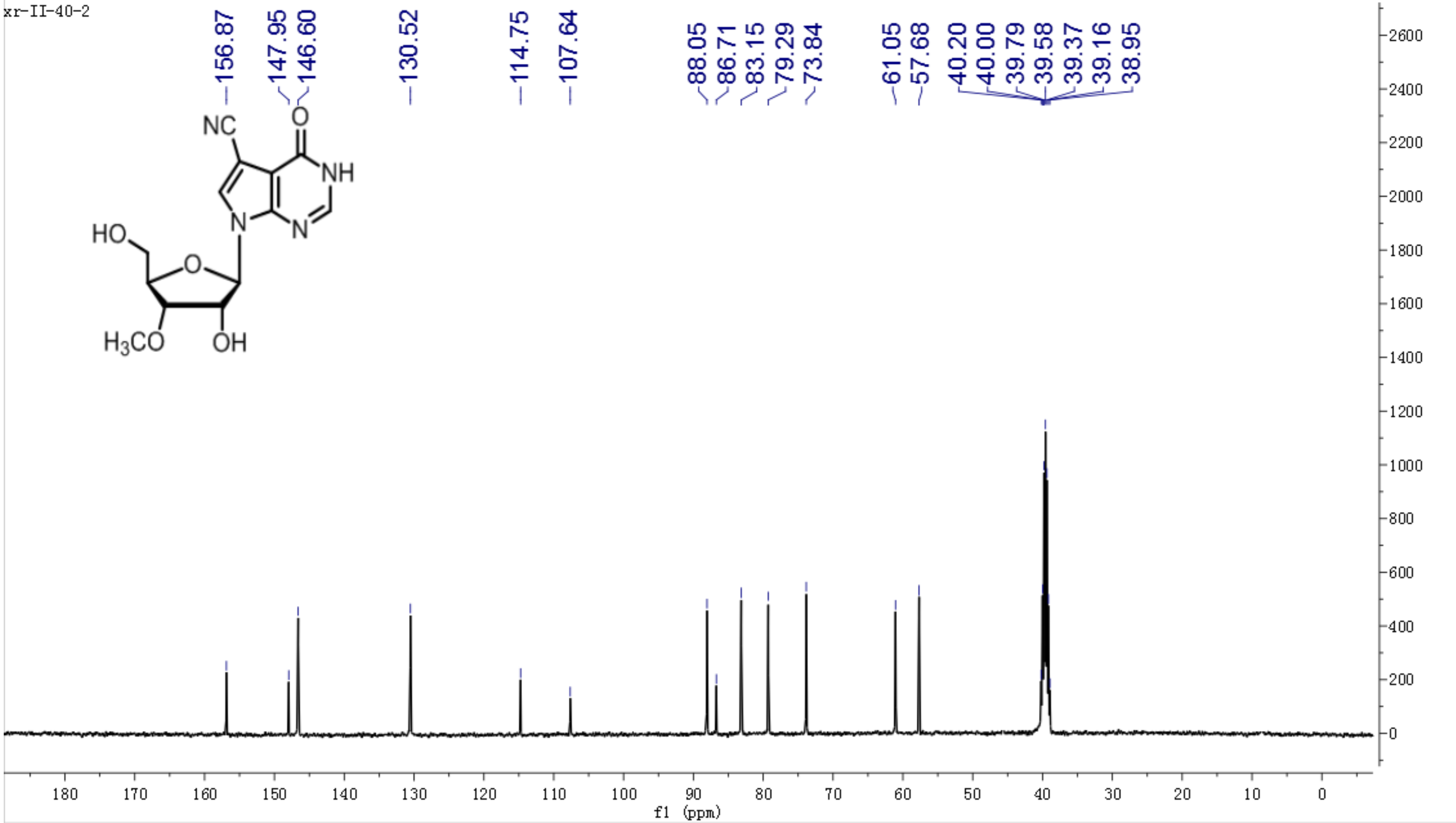

Fig.10. ^{1}H NMR and ^{13}C NMR of compound **20**

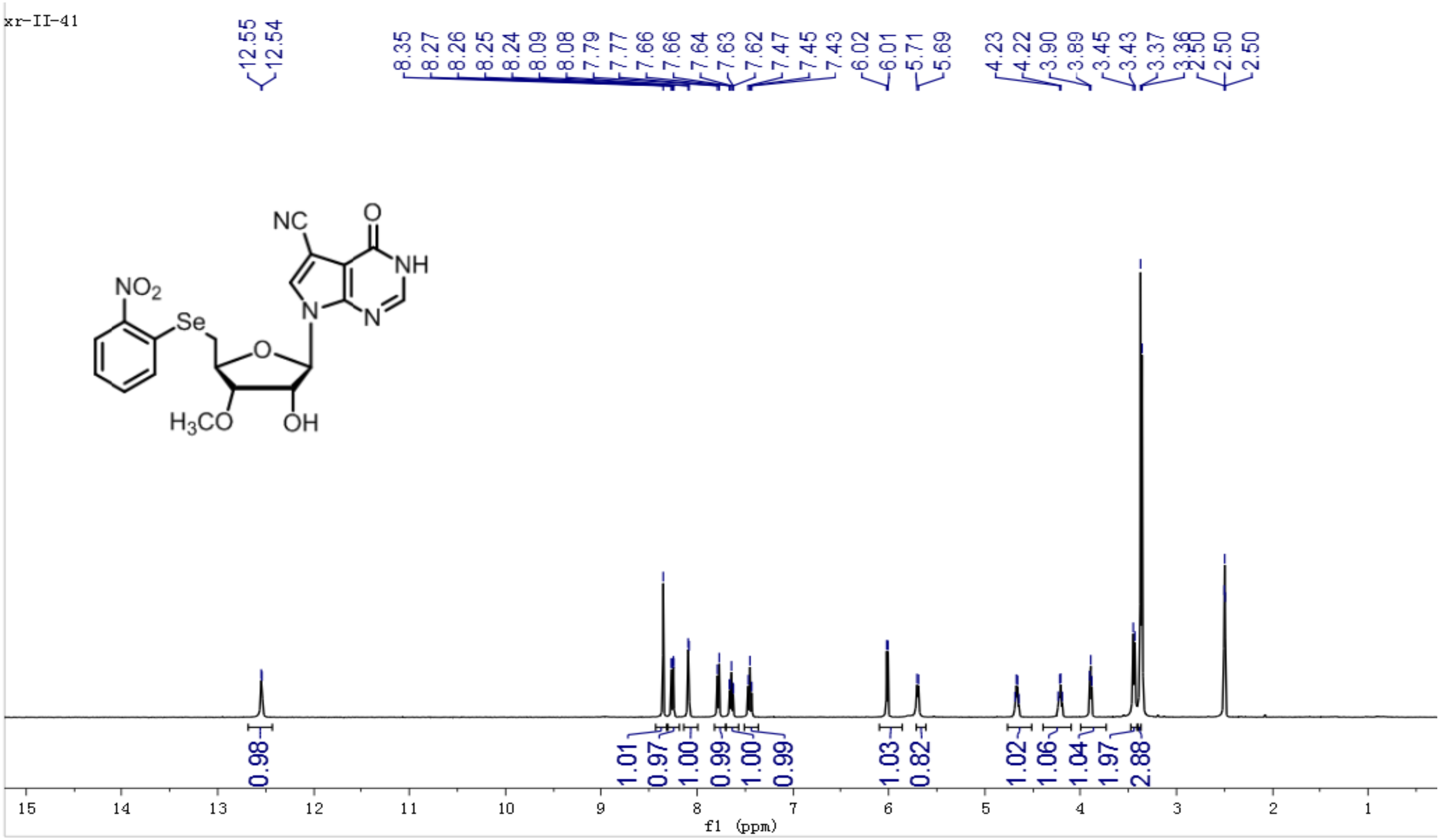

Fig.10. ^{1}H NMR and ^{13}C NMR of compound **20**

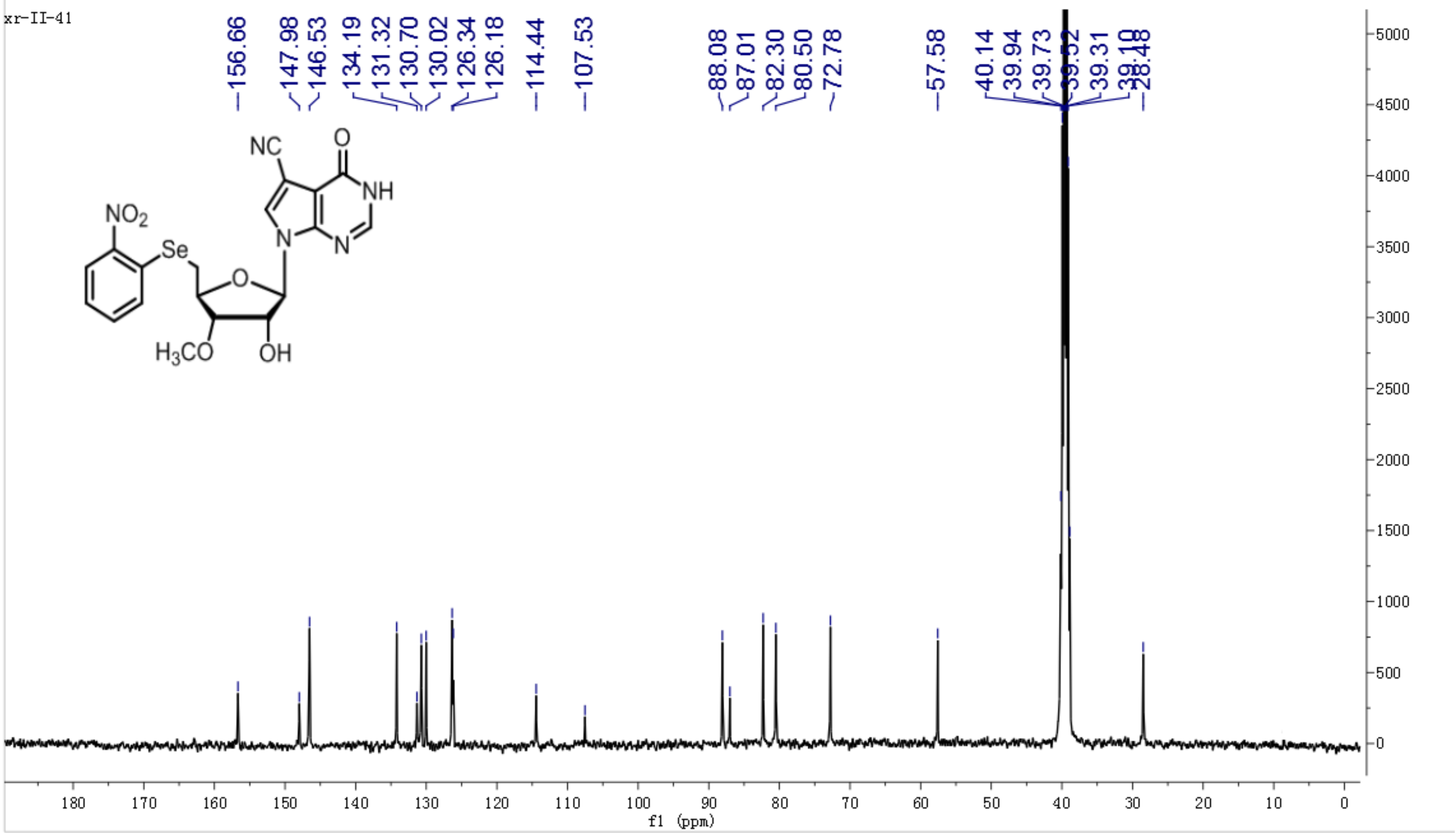

Crystallographic data of compound **11**

Table 1. Details of Data Collection, Processing and Structure Refinement

Identification code	**1**
Empirical formula	$C_{30} H_{36} O_{12}$
Formula weight	588.59
Temperature	296(2) K
Wavelength	0.71073 Å
Crystal system, space group	Monoclinic, P2(1)
Unit cell dimensions	a = 5.7106(8) Å alpha = 90°
	b = 16.631(2) Å beta = 91.331(2)°.
	c = 15.970(2) Å gamma = 90°.
Volume	1516.4(4) $Å^3$
Z, Calculated density	2, 1.289 mg/m^3
Absorption coefficient	0.100 mm^{-1}
F(000)	624
Crystal size	0.300 × 0.280 × 0.250 mm
Theta range for data collection	2.551 to 25.993 deg.
Limiting indices	-7< *h* <7, -20< *k* <14, -19< *l* <18
Reflections collected / unique	8381 / 4330 [R(int) = 0.0218]
Completeness to theta = 25.242	99.8 %
Absorption correction	None
Refinement method	Full-matrix least-squares on F^2
Data / restraints / parameters	4330 / 1 / 380
Goodness-of-fit on F^2	0.669
Final R indices [I>2sigma(I)]	R1 = 0.0351, wR2 = 0.1020
R indices (all data)	R1 = 0.0471, wR2 = 0.1189
Absolute structure parameter	0.1(6)
Extinction coefficient	0.015(3)
Largest diff. peak and hole	0.182 and -0.152 e. $Å^{-3}$

Table 2. Atomic coordinates (x 10^4) and equivalent isotropic temperature factors* ($Å^2 \times 10^3$) for **1**.

Atoms	x	y	z	U(eq)
C(1)	4517(9)	2737(3)	11308(4)	91(2)
C(2)	3173(10)	2798(4)	9786(4)	92(2)
C(3)	4551(6)	3192(2)	10489(3)	57(1)
C(4)	5506(5)	4507(2)	10811(2)	44(1)
C(5)	7727(6)	4057(2)	10555(2)	53(1)
C(6)	5397(5)	5230(2)	10213(2)	40(1)
C(7)	6943(5)	4956(2)	9500(2)	38(1)
C(8)	8017(6)	5638(2)	9033(2)	43(1)
C(9)	10907(6)	5828(2)	8020(2)	45(1)
C(10)	12716(6)	5427(2)	7509(2)	45(1)
C(11)	12908(7)	4594(3)	7475(2)	57(1)
C(12)	14647(8)	4247(3)	7005(3)	69(1)
C(13)	16186(7)	4724(3)	6575(3)	70(1)
C(14)	16017(7)	5542(3)	6613(2)	67(1)
C(15)	14273(6)	5905(3)	7077(2)	55(1)
C(16)	19(11)	2486(3)	4088(4)	91(2)
C(17)	-788(10)	3533(4)	2980(3)	94(2)
C(18)	392(6)	3346(2)	3810(2)	56(1)
C(19)	1365(5)	4183(2)	4935(2)	45(1)
C(20)	3594(6)	3972(2)	4474(2)	50(1)
C(21)	1281(5)	5097(2)	4920(2)	42(1)
C(22)	2809(5)	5305(2)	4175(2)	40(1)
C(23)	3889(6)	6125(2)	4224(2)	48(1)
C(24)	6717(6)	6864(2)	3489(2)	51(1)
C(25)	8418(6)	6826(2)	2797(2)	50(1)
C(26)	9793(8)	7497(3)	2641(3)	72(1)
C(27)	11382(9)	7481(4)	1992(3)	89(2)
C(28)	11582(8)	6804(4)	1516(3)	85(2)
C(29)	10284(8)	6138(4)	1677(3)	76(1)
C(30)	8687(7)	6147(3)	2321(2)	61(1)
O(1)	3639(4)	3976(2)	10605(2)	48(1)
O(2)	6899(5)	3321(2)	10226(2)	79(1)
O(3)	3128(4)	5464(2)	9955(2)	55(1)
O(4)	8783(3)	4510(1)	9923(2)	47(1)
O(5)	9599(4)	5299(1)	8435(1)	47(1)
O(6)	10628(6)	6545(2)	8064(2)	67(1)
O(7)	-493(4)	3887(2)	4418(2)	56(1)
O(8)	2837(5)	3521(2)	3777(2)	72(1)
O(9)	-991(4)	5433(2)	4860(2)	56(1)
O(10)	4619(4)	4703(2)	4224(2)	49(1)
O(11)	5397(4)	6203(2)	3518(1)	51(1)
O(12)	6531(6)	7417(2)	3970(2)	77(1)

*$U_{eq.}$ defined as one third of the trace of the orthogonalized **U** tensor.

Table 3. Bond lengths (Å) and bond angles (°) for **1**.

C(1)-C(3)	1.511(7)	C(7)-H(7A)	0.9800
C(1)-H(1A)	0.9600	C(8)-O(5)	1.444(4)
C(1)-H(1B)	0.9600	C(8)-H(8A)	0.9700
C(1)-H(1C)	0.9600	C(8)-H(8B)	0.9700
C(2)-C(3)	1.506(6)	C(9)-O(6)	1.205(5)
C(2)-H(2A)	0.9600	C(9)-O(5)	1.339(4)
C(2)-H(2B)	0.9600	C(9)-C(10)	1.489(5)
C(2)-H(2C)	0.9600	C(10)-C(11)	1.391(6)
C(3)-O(1)	1.419(4)	C(10)-C(15)	1.388(5)
C(3)-O(2)	1.431(4)	C(11)-C(12)	1.385(6)
C(4)-O(1)	1.417(4)	C(11)-H(11A)	0.9300
C(4)-C(5)	1.536(5)	C(12)-C(13)	1.379(7)
C(4)-C(6)	1.534(5)	C(12)-H(12A)	0.9300
C(4)-H(4A)	0.9800	C(13)-C(14)	1.365(7)
C(5)-O(4)	1.407(4)	C(13)-H(13A)	0.9300
C(5)-O(2)	1.410(5)	C(14)-C(15)	1.392(6)
C(5)-H(5A)	0.9800	C(14)-H(14A)	0.9300
C(6)-O(3)	1.405(4)	C(15)-H(15A)	0.9300
C(6)-C(7)	1.527(4)	C(16)-C(18)	1.514(7)
C(6)-H(6A)	0.9800	C(16)-H(16A)	0.9600
C(7)-O(4)	1.441(3)	C(16)-H(16B)	0.9600
C(7)-C(8)	1.497(4)	C(16)-H(16C)	0.9600
C(17)-C(18)	1.505(6)	C(24)-O(12)	1.205(5)
C(17)-H(17A)	0.9600	C(24)-O(11)	1.334(4)
C(17)-H(17B)	0.9600	C(24)-C(25)	1.489(5)
C(17)-H(17C)	0.9600	C(25)-C(30)	1.372(6)
C(18)-O(8)	1.429(5)	C(25)-C(26)	1.390(6)
C(18)-O(7)	1.424(4)	C(26)-C(27)	1.393(7)
C(19)-O(7)	1.418(4)	C(26)-H(26A)	0.9300
C(19)-C(21)	1.521(5)	C(27)-C(28)	1.366(8)
C(19)-C(20)	1.526(5)	C(27)-H(27A)	0.9300
C(19)-H(19A)	0.9800	C(28)-C(29)	1.360(7)
C(20)-O(8)	1.402(5)	C(28)-H(28A)	0.9300
C(20)-O(10)	1.411(4)	C(29)-C(30)	1.390(6)
C(20)-H(20A)	0.9800	C(29)-H(29A)	0.9300
C(21)-O(9)	1.414(4)	C(30)-H(30A)	0.9300
C(21)-C(22)	1.531(4)	O(3)-H(3A)	0.8200
C(21)-H(21A)	0.9800	O(9)-H(9A)	0.8200
C(22)-O(10)	1.440(4)	C(3)-C(1)-H(1A)	109.5
C(22)-C(23)	1.498(5)	C(3)-C(1)-H(1B)	109.5
C(22)-H(22A)	0.9800	H(1A)-C(1)-H(1B)	109.5
C(23)-O(11)	1.439(4)	C(3)-C(1)-H(1C)	109.5
C(23)-H(23A)	0.9700	H(1A)-C(1)-H(1C)	109.5

C(23)-H(23B)	0.9700	H(1B)-C(1)-H(1C)	109.5
C(3)-C(2)-H(2A)	109.5	O(2)-C(5)-H(5A)	111.5
C(3)-C(2)-H(2B)	109.5	C(4)-C(5)-H(5A)	111.5
H(2A)-C(2)-H(2B)	109.5	O(3)-C(6)-C(4)	115.0(3)
C(3)-C(2)-H(2C)	109.5	O(3)-C(6)-C(7)	114.1(2)
H(2A)-C(2)-H(2C)	109.5	C(4)-C(6)-C(7)	102.4(3)
H(2B)-C(2)-H(2C)	109.5	O(3)-C(6)-H(6A)	108.3
O(1)-C(3)-O(2)	104.5(3)	C(4)-C(6)-H(6A)	108.3
O(1)-C(3)-C(2)	108.0(3)	C(7)-C(6)-H(6A)	108.3
O(2)-C(3)-C(2)	108.8(4)	O(4)-C(7)-C(8)	108.7(2)
O(1)-C(3)-C(1)	109.6(4)	O(4)-C(7)-C(6)	103.4(2)
O(2)-C(3)-C(1)	111.1(3)	C(8)-C(7)-C(6)	113.3(3)
C(2)-C(3)-C(1)	114.3(4)	O(4)-C(7)-H(7A)	110.4
O(1)-C(4)-C(5)	104.9(3)	C(8)-C(7)-H(7A)	110.4
O(1)-C(4)-C(6)	108.9(2)	C(6)-C(7)-H(7A)	110.4
C(5)-C(4)-C(6)	103.8(3)	O(5)-C(8)-C(7)	107.6(3)
O(1)-C(4)-H(4A)	112.9	O(5)-C(8)-H(8A)	110.2
C(5)-C(4)-H(4A)	112.9	C(7)-C(8)-H(8A)	110.2
C(6)-C(4)-H(4A)	112.9	O(5)-C(8)-H(8B)	110.2
O(4)-C(5)-O(2)	110.1(3)	C(7)-C(8)-H(8B)	110.2
O(4)-C(5)-C(4)	107.5(3)	H(8A)-C(8)-H(8B)	108.5
O(2)-C(5)-C(4)	104.5(3)	O(6)-C(9)-O(5)	123.0(3)
O(4)-C(5)-H(5A)	111.5	O(5)-C(9)-C(10)	112.3(3)
C(11)-C(10)-C(15)	120.0(4)	H(16A)-C(16)-H(16C)	109.5
C(11)-C(10)-C(9)	121.6(3)	H(16B)-C(16)-H(16C)	109.5
C(15)-C(10)-C(9)	118.5(3)	C(18)-C(17)-H(17A)	109.5
C(10)-C(11)-C(12)	119.6(4)	C(18)-C(17)-H(17B)	109.5
C(10)-C(11)-H(11A)	120.2	H(17A)-C(17)-H(17B)	109.5
C(12)-C(11)-H(11A)	120.2	C(18)-C(17)-H(17C)	109.5
C(13)-C(12)-C(11)	120.2(5)	H(17A)-C(17)-H(17C)	109.5
C(13)-C(12)-H(12A)	119.9	H(17B)-C(17)-H(17C)	109.5
C(11)-C(12)-H(12A)	119.9	O(8)-C(18)-O(7)	105.0(3)
C(12)-C(13)-C(14)	120.3(4)	O(8)-C(18)-C(16)	110.4(4)
C(12)-C(13)-H(13A)	119.8	O(7)-C(18)-C(16)	110.0(4)
C(14)-C(13)-H(13A)	119.8	O(8)-C(18)-C(17)	110.1(4)
C(15)-C(14)-C(13)	120.5(4)	O(7)-C(18)-C(17)	108.1(3)
C(15)-C(14)-H(14A)	119.7	C(16)-C(18)-C(17)	112.9(4)
C(13)-C(14)-H(14A)	119.7	O(7)-C(19)-C(21)	108.4(3)
C(14)-C(15)-C(10)	119.4(4)	O(7)-C(19)-C(20)	105.1(3)
C(14)-C(15)-H(15A)	120.3	C(21)-C(19)-C(20)	104.4(3)
C(10)-C(15)-H(15A)	120.3	O(7)-C(19)-H(19A)	112.8
C(18)-C(16)-H(16A)	109.5	C(21)-C(19)-H(19A)	112.8
C(18)-C(16)-H(16B)	109.5	C(20)-C(19)-H(19A)	112.8
H(16A)-C(16)-H(16B)	109.5	C(18)-C(16)-H(16C)	109.5

C(18)-C(16)-H(16C)	109.5	O(8)-C(20)-O(10)	111.0(3)
O(8)-C(20)-C(19)	105.1(3)	H(23A)-C(23)-H(23B)	108.5
O(10)-C(20)-C(19)	107.1(3)	O(12)-C(24)-O(11)	123.3(3)
O(8)-C(20)-H(20A)	111.1	O(12)-C(24)-C(25)	125.0(3)
O(10)-C(20)-H(20A)	111.1	O(11)-C(24)-C(25)	111.7(3)
C(19)-C(20)-H(20A)	111.1	C(30)-C(25)-C(26)	119.3(4)
O(9)-C(21)-C(19)	115.1(3)	C(30)-C(25)-C(24)	122.0(3)
O(9)-C(21)-C(22)	113.4(3)	C(26)-C(25)-C(24)	118.7(4)
C(19)-C(21)-C(22)	102.7(3)	C(27)-C(26)-C(25)	119.9(5)
O(9)-C(21)-H(21A)	108.5	C(27)-C(26)-H(26A)	120.0
C(19)-C(21)-H(21A)	108.5	C(25)-C(26)-H(26A)	120.0
C(22)-C(21)-H(21A)	108.5	C(28)-C(27)-C(26)	119.6(5)
O(10)-C(22)-C(23)	109.6(3)	C(28)-C(27)-H(27A)	120.2
O(10)-C(22)-C(21)	102.8(3)	C(26)-C(27)-H(27A)	120.2
C(23)-C(22)-C(21)	114.0(3)	C(27)-C(28)-C(29)	120.9(4)
O(10)-C(22)-H(22A)	110.1	C(27)-C(28)-H(28A)	119.6
C(23)-C(22)-H(22A)	110.1	C(29)-C(28)-H(28A)	119.6
C(21)-C(22)-H(22A)	110.1	C(30)-C(29)-C(28)	120.0(5)
O(11)-C(23)-C(22)	107.1(3)	C(30)-C(29)-H(29A)	120.0
O(11)-C(23)-H(23A)	110.3	C(28)-C(29)-H(29A)	120.0
C(22)-C(23)-H(23A)	110.3	C(29)-C(30)-C(25)	120.2(4)
O(11)-C(23)-H(23B)	110.3	C(29)-C(30)-H(30A)	119.9
C(22)-C(23)-H(23B)	110.3	C(25)-C(30)-H(30A)	119.9
C(4)-O(1)-C(3)	109.1(3)	C(19)-O(7)-C(18)	110.1(2)
C(3)-O(2)-C(5)	109.2(3)	C(20)-O(8)-C(18)	111.3(3)
C(6)-O(3)-H(3A)	109.5	C(21)-O(9)-H(9A)	109.5
C(5)-O(4)-C(7)	107.0(2)	C(20)-O(10)-C(22)	108.2(2)
C(9)-O(5)-C(8)	115.8(3)	C(24)-O(11)-C(23)	116.8(3)

Symmetry transformations used to generate equivalent atoms

Table 4. Anisotropic thermal parameters* (Å^2)

Atoms	U11	U22	U33	U23	U13	U12
C(1)	65(3)	77(3)	131(4)	51(3)	-1(3)	-11(2)
C(2)	89(4)	77(4)	110(4)	-31(3)	14(3)	-15(3)
C(3)	40(2)	41(2)	90(3)	9(2)	15(2)	0(2)
C(4)	38(2)	51(2)	44(2)	2(1)	-1(1)	-7(2)
C(5)	34(2)	57(2)	69(2)	26(2)	-4(1)	-2(2)
C(6)	33(2)	36(2)	52(2)	-2(1)	0(1)	-3(1)
C(7)	31(1)	35(2)	48(2)	2(1)	-4(1)	2(1)
C(8)	42(2)	39(2)	48(2)	4(1)	2(1)	3(1)
C(9)	47(2)	47(2)	41(2)	6(1)	-2(1)	-7(2)
C(10)	43(2)	54(2)	37(1)	2(1)	-2(1)	-9(2)
C(11)	56(2)	60(2)	55(2)	-2(2)	5(2)	-6(2)
C(12)	68(3)	70(3)	69(2)	-12(2)	8(2)	6(2)
C(13)	53(2)	98(4)	59(2)	-14(2)	9(2)	0(2)
C(14)	53(2)	101(4)	48(2)	1(2)	5(2)	-22(2)
C(15)	57(2)	64(3)	43(2)	3(2)	2(2)	-13(2)
C(16)	98(4)	52(3)	122(4)	-10(3)	-1(3)	-4(3)
C(17)	99(4)	107(5)	76(3)	-14(3)	-19(3)	9(3)
C(18)	47(2)	51(2)	69(2)	-12(2)	-3(2)	0(2)
C(19)	36(2)	53(2)	46(2)	4(1)	-1(1)	-4(2)
C(20)	37(2)	46(2)	67(2)	0(2)	-2(1)	5(2)
C(21)	34(2)	53(2)	41(1)	-7(1)	2(1)	-2(1)
C(22)	31(1)	47(2)	43(2)	-6(1)	2(1)	5(1)
C(23)	46(2)	47(2)	50(2)	-2(2)	7(1)	1(2)
C(24)	60(2)	41(2)	52(2)	4(2)	-1(2)	-7(2)
C(25)	50(2)	50(2)	49(2)	5(2)	-1(1)	-5(2)
C(26)	76(3)	60(3)	82(3)	4(2)	13(2)	-16(2)
C(27)	80(3)	86(4)	102(3)	12(3)	28(3)	-31(3)
C(28)	65(3)	108(5)	82(3)	0(3)	21(2)	-17(3)
C(29)	66(3)	91(4)	70(2)	-17(2)	12(2)	-9(3)
C(30)	59(2)	64(3)	58(2)	-8(2)	6(2)	-13(2)
O(1)	33(1)	42(1)	70(1)	4(1)	3(1)	-2(1)
O(2)	47(2)	42(2)	151(3)	12(2)	37(2)	4(1)
O(3)	33(1)	49(2)	82(2)	7(1)	6(1)	7(1)
O(4)	27(1)	45(1)	69(1)	18(1)	3(1)	4(1)
O(5)	51(1)	42(1)	48(1)	2(1)	10(1)	-4(1)
O(6)	80(2)	43(2)	80(2)	12(1)	22(2)	-3(1)
O(7)	35(1)	55(2)	79(2)	-18(1)	-1(1)	-4(1)
O(8)	49(2)	74(2)	93(2)	-36(2)	15(1)	-5(1)
O(9)	33(1)	58(2)	79(2)	-18(1)	11(1)	3(1)
O(10)	30(1)	49(1)	69(1)	-1(1)	8(1)	7(1)
O(11)	55(1)	47(1)	50(1)	-2(1)	8(1)	-10(1)
O(12)	105(2)	50(2)	77(2)	-15(2)	30(2)	-16(2)

*The exponent takes the form: $-2\pi^2\Sigma\Sigma U_{ij}h_ih_j\mathbf{a}_i^*\mathbf{a}_j^*$

Table 5. Coordinates and isotropic temperature factors* ($Å^2 \times 10^3$) for H atoms

Atoms	x	y	z	U(eq)
H(1A)	5135	2207	11229	137
H(1B)	5459	3017	11720	137
H(1C)	2936	2700	11496	137
H(2A)	3762	2265	9696	138
H(2B)	1552	2770	9929	138
H(2C)	3327	3108	9283	138
H(4A)	5540	4665	11402	53
H(5A)	8800	3974	11035	64
H(6A)	6158	5688	10493	48
H(7A)	6059	4605	9114	46
H(8A)	6807	5949	8745	52
H(8B)	8863	5990	9418	52
H(11A)	11874	4272	7766	68
H(12A)	14778	3690	6978	83
H(13A)	17345	4488	6257	84
H(14A)	17076	5860	6328	81
H(15A)	14151	6462	7097	66
H(16A)	-1630	2376	4107	137
H(16B)	730	2126	3698	137
H(16C)	718	2409	4634	137
H(17A)	-2428	3413	3008	141
H(17B)	-585	4092	2853	141
H(17C)	-102	3212	2550	141
H(19A)	1349	3963	5503	54
H(20A)	4675	3661	4833	60
H(21A)	2056	5299	5432	51
H(22A)	1901	5248	3650	48
H(23A)	2682	6535	4202	57
H(23B)	4786	6185	4743	57
H(26A)	9653	7955	2970	87
H(27A)	12300	7929	1884	107
H(28A)	12619	6798	1076	102
H(29A)	10465	5677	1356	91
H(30A)	7798	5692	2429	73
H(3A)	2226	5085	10009	82
H(9A)	-1904	5101	4660	85

*The exponent takes the form: $-8\pi^2 U \sin^2\theta/\lambda$

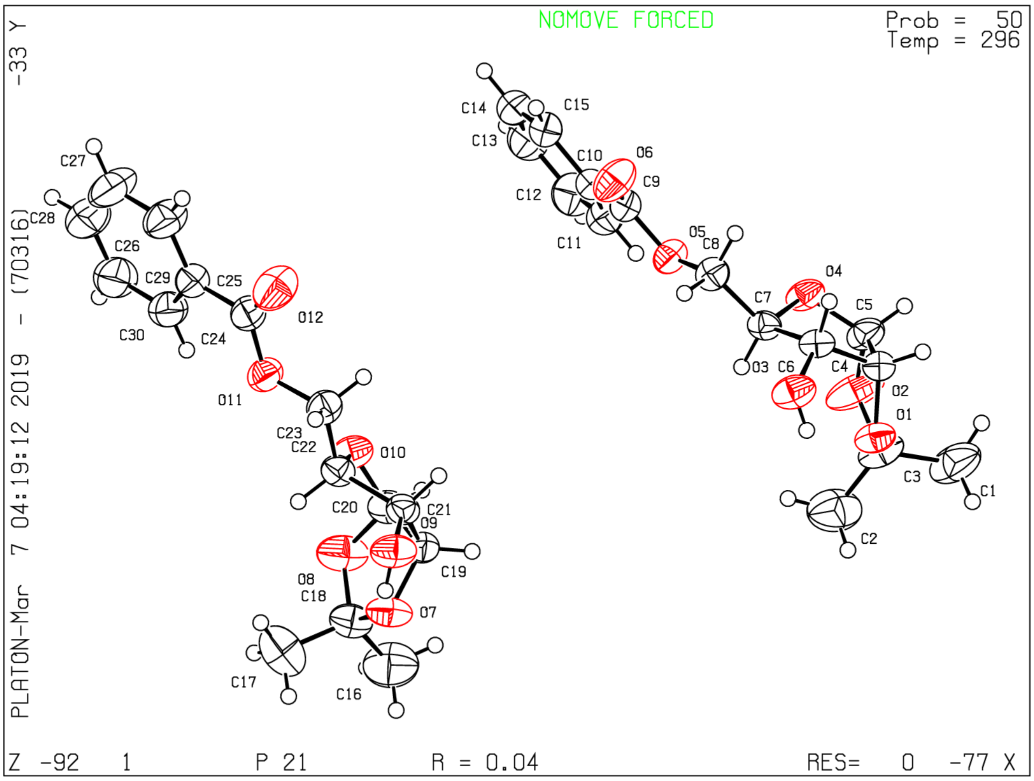
NOMOVE FORCED
Prob = 50
Temp = 296
-33 Y
PLATON-Mar 7 04:19:12 2019 - (70316)
Z -92 1
P 21
R = 0.04
RES= 0 -77 X
C14
C15
C13
C10
O6
C9
C12
O5
C8
C11
O4
C7
C5
O3
C6
C4
O2
O1
C3
C1
C2
C27
C28
C26
C29
C25
O12
C30
C24
O11
C23
C22
O10
C20
C21
O9
C19
O8
C18
O7
C17
C16
